# Influence of Ligand Backbone Structure and Connectivity on the Properties of Phosphine-Sulfonate Pd(II)/Ni(II) Catalysts

**DOI:** 10.3390/polym9050168

**Published:** 2017-05-09

**Authors:** Zixia Wu, Changwen Hong, Hongxu Du, Wenmin Pang, Changle Chen

**Affiliations:** Chinese Academy of Sciences Key Laboratory of Soft Matter Chemistry, Department of Polymer Science and Engineering, University of Science and Technology of China, Hefei 230026, China; wuzixia1@mail.ustc.edu.cn (Z.W.); Hongcw94@mail.ustc.edu.cn (C.H.); duhx14@mail.ustc.edu.cn (H.D.); pangwm@ustc.edu.cn (W.P.)

**Keywords:** phosphine-sulfonate, palladium, nickel, olefin polymerization, copolymerization

## Abstract

Phosphine-sulfonate based palladium and nickel catalysts have been extensively studied in ethylene polymerization and copolymerization reactions. Previously, the majority of the research works focused on the modifications of the substituents on the phosphorous atom. In this contribution, we systematically demonstrated that the change of the ligand backbone from benzene to naphthalene could greatly improve the properties of this class of catalysts. In the palladium system, this change could increase catalyst stability and polyethylene molecular weights. In the nickel system, this change could dramatically increase the polyethylene molecular weights. Most interestingly, the change in the connectivity of phosphine and sulfonate moieties to the naphthalene backbone could also significantly influence the catalyst properties.

## 1. Introduction

**Scheme 1:**
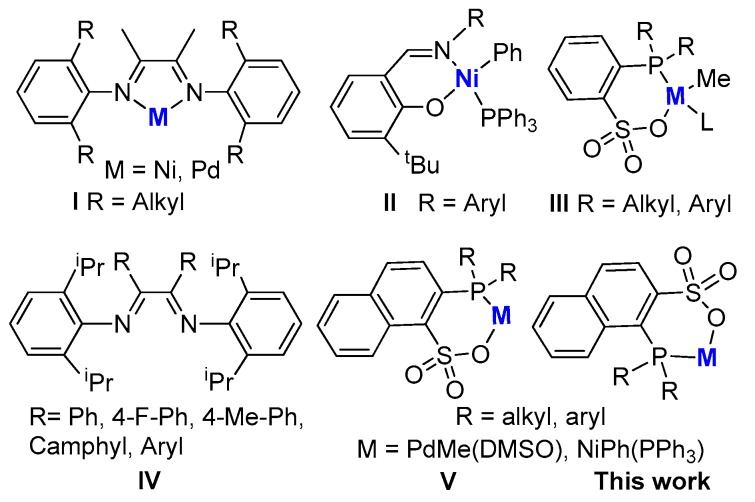
The α-diimine, phenoxyminato and phosphine-sulfonate based olefin polymerization catalysts.

In olefin polymerization, late transition metal catalysts have attracted much attention because of their low oxophilicity, and correspondingly the potentials to incorporation polar functionalized monomers into polyolefins. Among the numerous late transition metal catalysts, the Brookhart type α-diimine Ni(II) and Pd(II) [1,2,3,4,5,6,7,8,9,10,11,12,13,14], phenoxyminato based Ni(II) [15,16,17,18,19,20,21] and the phosphine-sulfonate Pd(II) catalysts [22,23,24,25,26,27,28,29,30,31,32,33,34,35] are the most extensively studied systems. It has been demonstrated that the properties of these catalysts are very sensitive to the ligand sterics. Specifically, it has been well established that the steric bulk on the axial positions in α-diimine systems could decrease the chain transfer rate, and increase the polyolefin molecular weight (Scheme 1, **I**) [1,2,36,37,38,39]. Similarly, the steric bulk on the axial positions in phenoxyminato (Scheme 1, **II**) and phosphine-sulfonate (Scheme 1, **III**) systems is crucial to obtain high-performance catalysts [40,41,42].

The modulation of the steric effect on the axial positions directly affects the steric environment of the metal center. In addition, the ligand backbone structure could indirectly influence the steric environment of the metal center, and correspondingly affect the properties of the metal catalysts. For example, different substituents on the backbone R position could greatly alter the properties of the α-diimine Pd(II) and Ni(II) catalysts (Scheme 1, **IV**) [43,44,45]. Despite the various efforts to modify the phosphine-sulfonate ligands, there have been very few studies on the modifications of the ligand backbone structures [46]. Recently, our group showed that the catalyst stability and activity could be greatly enhanced by changing the phosphine-sulfonate backbone from a benzene bridge to a naphthalene one (Scheme 1, **V**) [47]. In this contribution, we hope to further improve the properties of the naphthalene based phosphine-sulfonate Pd(II) and Ni(II) catalysts by: (1) changing the linking position of the phosphine and the sulfonate moieties on the naphthalene backbone; and (2) using a sterically very bulky bi-aryl substituent on the phosphorous atom.

## 2. Results and Discussion

**Scheme 2:**
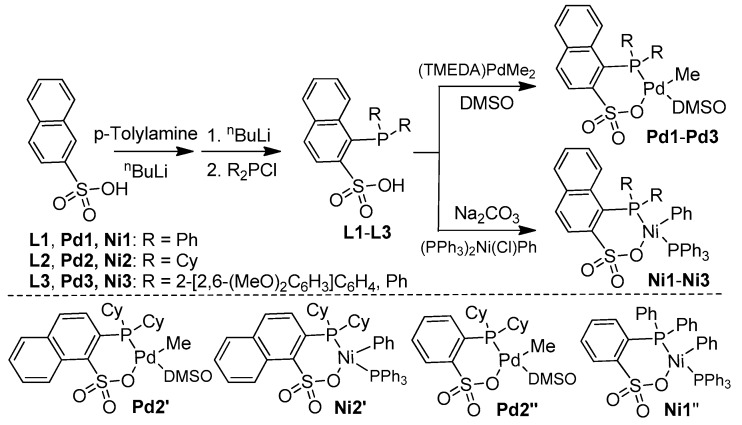
Synthesis of the phosphine-sulfonate ligands and the palladium and nickel complexes.

Literature procedure was employed to prepare the ligands [47]. First, the 2-naphthalenesulfonic acid was converted to the toluidinium salt from the reaction with excess amount of *p*-toluidine (Scheme 2, see Supplementary Materials, Experimental Sections). The corresponding lithium salt was generated from the reaction with 1 equivalent of *^n^*BuLi, and dehydrated using a dean-stark apparatus in refluxing toluene. Subsequently, ligands **L1**-**L3** were obtained in 39–47% yields from the reaction of 1 equivalent of *^n^*BuLi with the lithium salt in THF (tetrahydrofuran) followed by the addition of R_2_PCl. These ligands were characterized by by ^1^H, ^13^C, and ^31^P NMR (Nuclear Magnetic Resonance) spectroscopy (Bruker, Karlsruhe, Germany) (see Supplementary Materials, Figures S1–S9), elemental analysis and Mass spectrometry (EI+, Bruker Daltonics Inc., Billerica, MA, USA).

The reactions of ligands **L1**-**L3** with (TMEDA)PdMe_2_ (TMEDA = tetramethylethylenediamine) in DMSO (dimethylsulfoxide) led to the formation of the Pd(II) complexes (**Pd1**-**Pd3**) at 41–48% yields (Scheme 2). The reaction of ligands **L1**-**L3** with Na_2_CO_3_ and *trans*-[(PPh_3_)_2_Ni(Cl)Ph] afforded the desired Ni(II) complexes (**Ni1**-**Ni3**) at 34–76% yields. These metal complexes were characterized using ^1^H, ^13^C, and ^31^P NMR (see Supplementary Materials, Figures S10–S20), elemental analysis and MALDI-TOF-MS (Matrix Assisted Laser Desorption Ionization-Time of Flight-Mass Spectrometry). For comparison purpose, the palladium complex **Pd2′**/**Pd2″** and the nickel complex **Ni2′**/**Ni1″** were prepared according to literature procedures [48,49].

**Figure 1:**
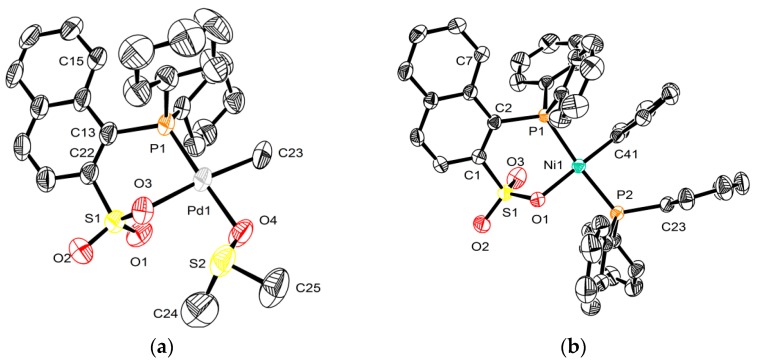
Molecular structures of: (**a**) **Pd2**; and (**b**) **Ni1**. Hydrogen atoms have been omitted for clarity. Selected bond lengths (Å) and angles (°) for **Pd2**: Pd1-C23 = 2.067(11), Pd1-P1 = 2.211(2), Pd1-O3 = 2.143(7), Pd1-O4 = 2.131(7), S1-O3 = 1.469(8), S1-O1 = 1.436(8), S1-C22 = 1.781(9), P1-C13 = 1.864(3), C13-C22 = 1.387(4), P1-Pd1-O32 = 91.3(1), P1-Pd1-O4 = 177.3(2), P1-Pd1-C23 = 94.7(3), Pd1-P1-C13 = 115.5(3), S1-O3-Pd1 = 112.9(4); for **Ni1**: Ni1-C41 = 1.8873(15), Ni1-P1 = 2.2221(4), Ni1-P2 = 2.2100(4), Ni1-O1 = 1.9553(11), S1-O1 = 1.4352(13), P2-C23 = 1.8309(15), P2-C17 = 1.8118(17), P1-Ni1-O1 = 93.63(3), P1-Ni1-C41 = 88.18(5), P2-Ni1-O1 = 89.47(3), P2-Ni1-C41 = 89.62(5), O1-Ni1-C41 = 174.92(6).

The molecular structures of **Pd2** and **Ni1** were determined by X-ray diffraction analysis (Figure 1; see CIF files and Supplementary Materials, Tables S1 and S2). The geometry at both the palladium and the nickel center is square planar with the methyl or phenyl substituent *cis* to the phosphine group. Clearly, the hydrogen atom on the C15 in **Pd2** and C7 in **Ni1** could exert some steric influence to the substituents on the phosphorous atom. Especially, this steric effect could be enhanced when the substituents are bulky. Most importantly, this interaction could potentially influence the properties of these catalysts in ethylene polymerization and copolymerization reactions.

The palladium catalysts are highly active in ethylene polymerization, with activities well above 10^5^ g·mol^−1^·h^−1^ (Table 1, entries 1–6). Catalyst **Pd3** with the biaryl substituent showed almost 10-fold increase in polymer molecular weight comparing with catalyst **Pd1**. The nickel catalysts are also highly active in ethylene polymerization, with activities comparable with those of the palladium catalysts (Table 1, entries 7–9). The palladium catalysts completely lost activity at 25 °C. However, the nickel catalysts could maintain high activity at 25 °C. Most importantly, the polyethylene molecular weight could be dramatically increased at lower polymerization temperature. For the case of **Ni3**, molecular weight of up to 142,300 could be achieved (Table 1, entry 12). Similar with the palladium case, catalyst **Ni3** with the biaryl substituent showed much higher polymer molecular weight than catalysts **Ni1** and **Ni2**.

**Figure 2:**
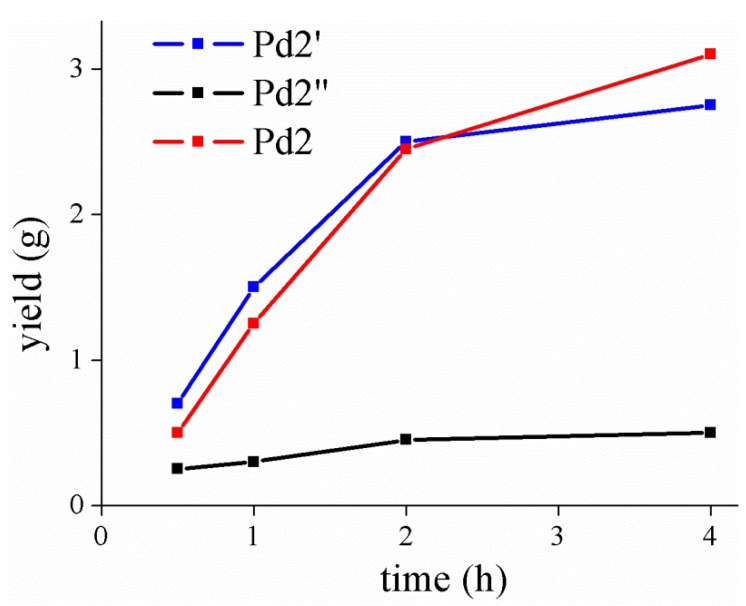
Polyethylene yield versus polymerization time at 80 °C for catalysts **Pd2**, **Pd2′** and **Pd2″**.

Some very interesting results were obtained from the comparisons between catalysts **Pd2**/**Ni2** and our previously reported catalysts **Pd2′**/**Ni2′**. Catalyst **Pd2** showed similar activity and similar polymer molecular weight with catalyst **Pd2′** (Table 1, entry 2 versus 13). The polyethylene molecular weights for catalysts **Pd2** and **Pd2′** are much higher than that of the conventional catalyst **Pd2″** with the benzene as ligand backbone. In terms of catalyst stability, catalyst **Pd2** showed slightly better stability than catalyst **Pd2′**, both of which are much more stable than conventional catalyst **Pd2″** (Figure 2). Clearly, the change in the ligand backbone from benzene to naphthalene could significantly improve the performance of phosphine-sulfonate palladium catalysts. However, the change in the connectivity of the phosphine moiety and the sulfonate moiety to the naphthalene backbone does not influence the properties of these palladium catalysts.

In the nickel system, catalyst **Ni2** showed polyethylene molecular weight twice as much as that of catalyst **Ni2′** (Table 1, entry 8 versus 14, entry 11 versus 15). This suggested that the very small perturbations in ligand sterics could exert significant effect on the properties of the phosphine-sulfonate nickel catalysts. Most interestingly, more dramatically differences were observed for the cases of catalyst **Ni1** versus catalyst **Ni1″** bearing phenyl substituent on the phosphorous atom. No isolate solid polymer product was generated by catalyst **Ni1″** in ethylene polymerization at either 80 or 25 °C. This agrees well with literature results [9]. In contrast, catalyst **Ni1** demonstrated high activity (up to 1.9 × 10^5^ g·mol^−1^·h^−1^), high polymer molecular weight (up to 37,200) and high melting temperature (134 °C) in ethylene polymerization. Cleary, the ligand backbone structure also plays an important role in determining the catalyst properties.

The palladium catalysts **Pd1**-**Pd3** can also initiate efficient ethylene/metal acrylate copolymerization, with comonomer incorporation ratios ranging between 3% and 27% (Table 2, entries 1–6). The polymer molecular weights were dramatically reduced comparing with those in ethylene homopolymerization. Because of the great performance of catalyst **Ni3** in ethylene homopolymerization, its properties in ethylene/polar monomer copolymerization were also investigated. Recently, Coates et al. showed that α-diimine nickel catalyst could mediate ethylene/methyl 10-undecenoate copolymerization in the presence of MAO (methylaluminoxane) [50]. Our groups showed that some sterically very bulky phosphine-sulfonate nickel catalysts could copolymerize ethylene with various polar monomers [51]. Here, **Ni3** could also achieve moderate catalytic activity, along with moderate comonomer incorporation and high copolymer molecular weights in ethylene copolymerization with methyl 10-undecenoate and 6-chloro-1-hexene (Table 2, entries 7 and 8). The palladium complex **Pd2″** with benzene backbone showed much lower activity and copolymer molecular weight than the corresponding complex **Pd2** with the naphthalene backbone (Table 2, entries 9 and 10). Moreover, the nickel complex **Ni1″** with benzene backbone is not active in the copolymerization (Table 2, entry 11).

**Scheme 3:**
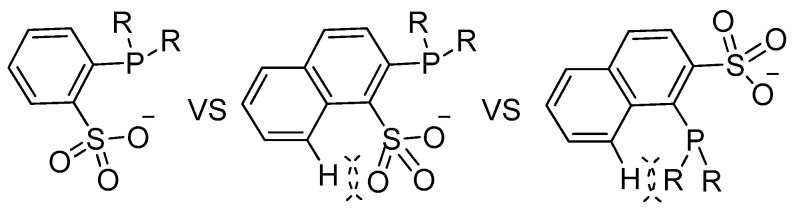
Comparison of the three ligand frameworks.

Clearly, great enhancement in the polymerization properties was achieved by changing the ligand backbone from benzene to naphthalene (Scheme 3). Ligand electronic effect may play an important role. 1-Naphthalenesulfonic acid (pKa = 0.17 at 298 K in aqueous solution) is more acidic than benzenesulfonic acid (pKa = 0.70 at 298 K in aqueous solution) [52], suggesting that naphthalene based ligand is electronically more withdrawing than the benzene based ligand. Moreover, the bigger size of the naphthalene backbone may help to prevent catalyst deactivation reactions such as bis-ligation [53]. Furthermore, the potential interaction of the hydrogen atom on the 8 position of the naphthalene backbone with the phosphine or sulfonate substituent may also influence the catalyst properties. This steric effect may be the key factor in the differences between the two sets of catalysts with different connectivity to the naphthalene backbone.

## 3. Conclusions

To conclude, a series of phosphine-sulfonate based palladium and nickel catalysts were prepared and characterized. A naphthalene bridge was installed in the ligand framework. These palladium and nickel catalysts showed very high activities in ethylene polymerization. For the case of nickel system, very high polyethylene molecular weights could be achieved. Both the palladium and the nickel catalysts could initiate ethylene-polar monomer copolymerization. We clearly demonstrated the importance of ligand backbone structure and connectivity in determining the properties of these metal catalysts. This work provides an alternative strategy to modify/improve the properties of group 10 phosphine-sulfonate catalysts. 

## Figures and Tables

**Table 1:** Ethylene polymerization catalyzed by Pd(II) and Ni(II) complexes ^a^.

Entry	Catalyst	[cat] (μmol)	*T* (°C)	Yield (g)	Activity ^b^	*M_w_* ^c^	Polydispersity ^c^	*T_m_* (°C) ^d^
1	**Pd1**	10	80	2	2.0	2100	1.15	109
2	**Pd2**	10	80	2.4	2.4	5100	2.36	117
3	**Pd3**	10	80	5	5.0	27,800	2.56	129
4	**Pd1**	2	80	0.5	2.5	3200	2.06	114
5	**Pd2**	2	80	0.4	2.0	5700	2.18	124
6	**Pd3**	2	80	1.2	6.0	24,800	1.84	131
7	**Ni1**	10	80	1.9	1.9	3600	1.58	128
8	**Ni2**	10	80	2.2	2.2	9100	2.18	129
9	**Ni3**	10	80	5.7	5.7	17,500	2.04	133
10	**Ni1**	2	25	0.7	1.7	37,200	3.64	134
11	**Ni2**	2	25	0.5	1.3	58,600	1.99	135
12	**Ni3**	2	25	1.4	3.5	142,300	1.51	136
13	**Pd2′**	10	80	2.1	2.1	4200	2.28	123
14	**Pd2″**	10	80	1.4	1.4	1800	1.44	115
15	**Ni2′**	10	80	1.5	1.5	4100	1.46	124
16	**Ni2′**	2	25	0.4	2.0	27,800	2.57	133
17	**Ni1″**	10	80	trace	-	-	-	-
18	**Ni1″**	2	25	trace	-	-	-	-

^a^ Polymerization conditions: toluene = 22 mL, CH_2_Cl_2_ = 3 mL, ethylene = 9 atm, 80 °C, time = 1 h. ^b^ Activity is in unit of 10^5^ g·mol^−1^·h^−1^. ^c^ Determined by Gel Permeation Chromatograph (GPC) in trichlorobenzene at 150 °C (see Supplementary Materials, Figures S36–S49). ^d^ Melting temperature was determined by differential scanning calorimetry (DSC) (see Supplementary Materials, Figures S26–S35).

**Table 2:** Ethylene copolymerization catalyzed by Pd(II) and Ni(II) complexes ^a^.

Entry	Catalyst	*P* (bar)	*T* (^o^C)	Comonomer	[*M*] mol/L	Yield (mg)	Activity ^b^	*X* ^c^ (%)	*M_w_* ^d^	Polydispersity ^d^
1	**Pd1**	9	80	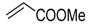	1.2	500	25	3	1000	1.19
2	**Pd2**	9	80	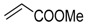	1.2	340	17	5	4400	1.92
3	**Pd3**	9	80	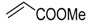	1.2	950	47	15	5500	1.55
4	**Pd1**	9	80	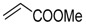	2.5	200	10	8	1600	1.43
5	**Pd2**	9	80	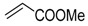	2.5	300	15	12	2100	1.35
6	**Pd3**	9	80	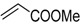	2.5	510	25	27	3600	1.58
7	**Ni3**	9	25	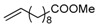	1.0	150	7.5	1.5	61,500	2.64
8	**Ni3**	9	25	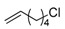	1.0	500	25	0.5	124,600	2.25
9	**Pd2″**	9	25	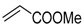	1.2	110	5.5	2.5	1950	1.95
10	**Pd2″**	9	25	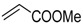	2.5	80	4	6	1100	1.63
11	**Ni1″**	9	25	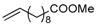	1.0	0	0	-	-	-

^a^ Polymerization conditions: total volume toluene + polar monomer = 25 mL, catalyst = 20 μmol. ^b^ Activity in unit of 10^3^ g·mol^−1^·h^−1^. ^c^ Amount of polar monomer incorporated (mol %), determined by ^1^H NMR spectroscopy (see Supplementary Materials, Figures S21–S25). ^d^ Determined by GPC in trichlorobenzene at 150 °C (see Supplementary Materials, Figures S49–S56).

## Supplementary Materials

The following are available online at www.mdpi.com/2073-4360/9/5/168/s1. Experimental procedures, characterization for ligands, palladium complexes, nickel complexes (Figures S1–S20), polyethylene and copolymers (Figures S21–S56), and CIF files for complexes Pd2 and Ni1.
