# Supplementary material for: Influence of Ligand Backbone Structure and Connectivity on the Properties of Phosphine-Sulfonate Pd(II)/Ni(II) Catalysts
Source: Polymers (Basel). 2017 May 9;9(5):168. doi: 10.3390/polym9050168 (PMC6431890; doi:10.3390/polym9050168)

checkCIF/PLATON report

Structure factors have been supplied for datablock(s) shelxl

THIS REPORT IS FOR GUIDANCE ONLY. IF USED AS PART OF A REVIEW PROCEDURE FOR PUBLICATION, IT SHOULD NOT REPLACE THE EXPERTISE OF AN EXPERIENCED CRYSTALLOGRAPHIC REFEREE.

No syntax errors found. CIF dictionary Interpreting this report

Datablock: shelxl

```
Bond precision:  C-C = 0.0170 A          Wavelength=1.54184

Cell:           a=32.7912(14)     b=11.1661(4)       c=15.8654(5)
                alpha=90          beta=101.751(4)    gamma=90
Temperature:    290 K

                Calculated                 Reported
Volume          5687.4(4)                  5687.4(4)
Space group     C 2/c                      C 2/c
Hall group      -C 2yc                     -C 2yc
Moiety formula  C25 H37 O4 P Pd S2         ?
Sum formula     C25 H37 O4 P Pd S2         C25 H37 O4 P Pd S2
Mr              603.04                     603.03
Dx,g cm-3       1.409                      1.409
Z               8                          8
Mu (mm-1)       7.386                      7.386
F000            2496.0                     2496.0
F000'           2510.10
h,k,lmax        40,13,19                   39,13,19
Nref            5462                       5373
Tmin,Tmax       0.133,0.158                0.073,1.000
Tmin'           0.037

Correction method= # Reported T Limits: Tmin=0.073 Tmax=1.000
AbsCorr = MULTI-SCAN

Data completeness= 0.984          Theta(max)= 70.637

R(reflections)= 0.1079( 4233)     wR2(reflections)= 0.2703( 5373)

S = 1.078                  Npar= 301
```

The following ALERTS were generated. Each ALERT has the format
test-name_ALERT_alert-type_alert-level.
Click on the hyperlinks for more details of the test.

Alert level B

```
PLAT601_ALERT_2_B Structure Contains Solvent Accessible VOIDS of .        172 Ang3
```

Alert level C

```
RINTA01_ALERT_3_C  The value of Rint is greater than 0.12
            Rint given   0.126
PLAT020_ALERT_3_C The value of Rint is greater than 0.12 .........      0.126 Report
PLAT082_ALERT_2_C High R1 Value ..................................       0.11 Report
PLAT084_ALERT_3_C High wR2 Value (i.e. > 0.25) ...................       0.27 Report
PLAT234_ALERT_4_C Large Hirshfeld Difference S2     --  C24     ..       0.22 Ang.
PLAT241_ALERT_2_C High   'MainMol' Ueq as Compared to Neighbors of         C3 Check
PLAT242_ALERT_2_C Low    'MainMol' Ueq as Compared to Neighbors of         S2 Check
PLAT342_ALERT_3_C Low Bond Precision on  C-C Bonds ...............    0.01704 Ang.
PLAT410_ALERT_2_C Short Intra H...H Contact   H1      ..  H15     .       1.96 Ang.
PLAT911_ALERT_3_C Missing # FCF Refl Between THmin & STh/L=  0.600          3 Report
PLAT971_ALERT_2_C Check Calcd Residual Density  1.00A From     Pd1       2.14 eA-3
PLAT971_ALERT_2_C Check Calcd Residual Density  1.23A From     Pd1       2.11 eA-3
PLAT971_ALERT_2_C Check Calcd Residual Density  0.99A From     Pd1       2.05 eA-3
PLAT971_ALERT_2_C Check Calcd Residual Density  1.03A From     Pd1       1.89 eA-3
PLAT971_ALERT_2_C Check Calcd Residual Density  1.04A From     Pd1       1.83 eA-3
PLAT971_ALERT_2_C Check Calcd Residual Density  1.08A From     Pd1       1.81 eA-3
PLAT971_ALERT_2_C Check Calcd Residual Density  0.98A From     Pd1       1.78 eA-3
PLAT971_ALERT_2_C Check Calcd Residual Density  0.95A From     Pd1       1.72 eA-3
PLAT972_ALERT_2_C Check Calcd Residual Density  1.84A From     Pd1      -1.62 eA-3
PLAT972_ALERT_2_C Check Calcd Residual Density  0.88A From     Pd1      -1.54 eA-3
PLAT973_ALERT_2_C Check Calcd Positive Residual Density on     Pd1       1.41 eA-3
PLAT975_ALERT_2_C Check Calcd Residual Density  1.08A From      O3       0.77 eA-3
PLAT978_ALERT_2_C Number C-C Bonds with Positive Residual Density.          0 Note
```

Alert level G

```
PLAT083_ALERT_2_G SHELXL Second Parameter in WGHT  Unusually Large      95.25 Why ?
PLAT910_ALERT_3_G Missing # of FCF Reflection(s) Below Theta(Min)           1 Note
PLAT912_ALERT_4_G Missing # of FCF Reflections Above STh/L=  0.600         84 Note
```

---

 0 **ALERT level A** = Most likely a serious problem - resolve or explain
 1 **ALERT level B** = A potentially serious problem, consider carefully
23 **ALERT level C** = Check. Ensure it is not caused by an omission or oversight
 3 **ALERT level G** = General information/check it is not something unexpected

 0 ALERT type 1 CIF construction/syntax error, inconsistent or missing data
19 ALERT type 2 Indicator that the structure model may be wrong or deficient
 6 ALERT type 3 Indicator that the structure quality may be low
 2 ALERT type 4 Improvement, methodology, query or suggestion
 0 ALERT type 5 Informative message, check

It is advisable to attempt to resolve as many as possible of the alerts in all categories. Often the minor alerts point to easily fixed oversights, errors and omissions in your CIF or refinement strategy, so attention to these fine details can be worthwhile. In order to resolve some of the more serious problems it may be necessary to carry out additional measurements or structure refinements. However, the purpose of your study may justify the reported deviations and the more serious of these should normally be commented upon in the discussion or experimental section of a paper or in the "special_details" fields of the CIF. checkCIF was carefully designed to identify outliers and unusual parameters, but every test has its limitations and alerts that are not important in a particular case may appear. Conversely, the absence of alerts does not guarantee there are no aspects of the results needing attention. It is up to the individual to critically assess their own results and, if necessary, seek expert advice.

Publication of your CIF in IUCr journals

A basic structural check has been run on your CIF. These basic checks will be run on all CIFs submitted for publication in IUCr journals (*Acta Crystallographica*, *Journal of Applied Crystallography*, *Journal of Synchrotron Radiation*); however, if you intend to submit to *Acta Crystallographica Section C* or *E* or *IUCrData*, you should make sure that full publication checks are run on the final version of your CIF prior to submission.

Publication of your CIF in other journals

Please refer to the *Notes for Authors* of the relevant journal for any special instructions relating to CIF submission.

PLATON version of 27/03/2017; check.def file version of 24/03/2017

Datablock shelxl - ellipsoid plot

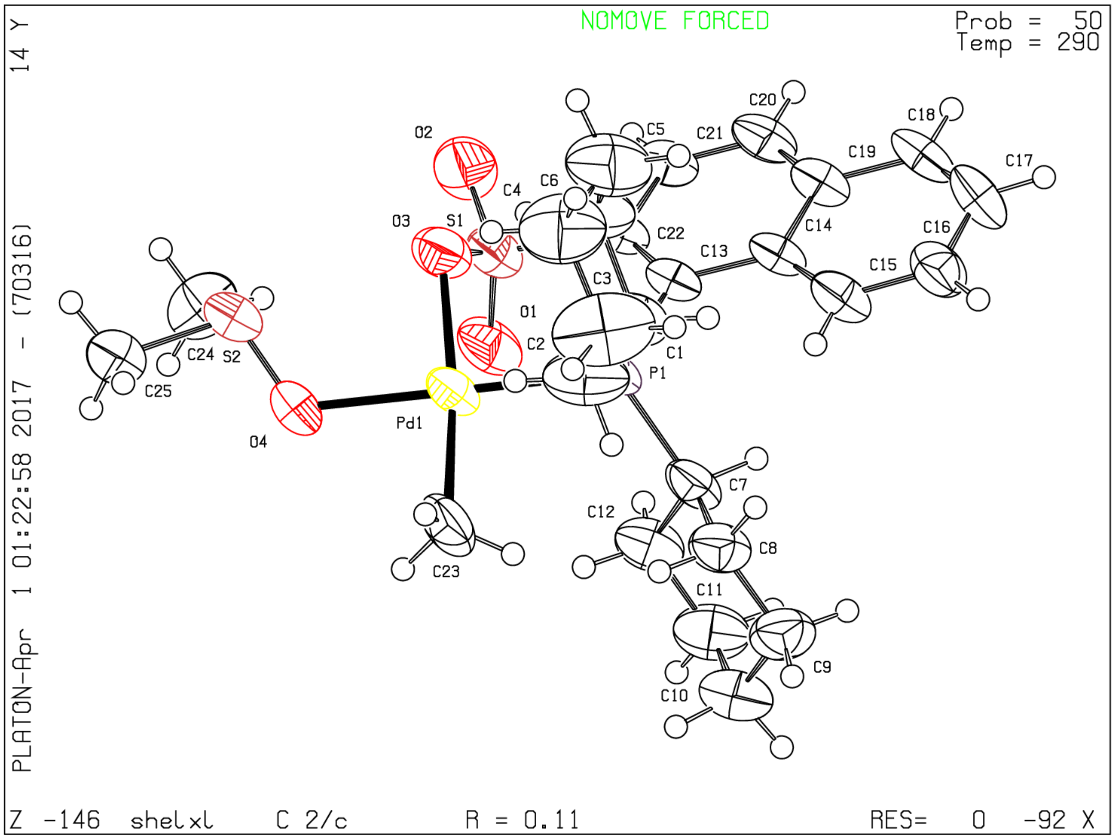

Supplement: Supplementary file 1 [file polymers-09-00168-s001.zip › checkcif-Pd.pdf]
