# Supplementary material for: Influence of Ligand Backbone Structure and Connectivity on the Properties of Phosphine-Sulfonate Pd(II)/Ni(II) Catalysts
Source: Polymers (Basel). 2017 May 9;9(5):168. doi: 10.3390/polym9050168 (PMC6431890; doi:10.3390/polym9050168)
Supplement: Supplementary file 1 [file polymers-09-00168-s001.zip › polymers-09-00168-s001.pdf]

polymers

Supporting Information

1. Experimental Sections

General Considerations

All experiments were carried out under a dry nitrogen atmosphere using standard Schlenk techniques or in a glove-box. Deuterated solvents used for NMR were dried and distilled prior to use. ^{1}H, ^{13}C and ^{31}P NMR spectra were recorded using a Bruker Ascend™ 400 spectrometer at an ambient temperature unless otherwise stated. The chemical shifts of the ^{1}H and ^{13}C NMR spectra were referenced to TMS; the ^{31}P NMR spectra were referenced to external 85% H_3PO_4. Coupling constants are in Hz. X-ray diffraction data were collected at 298 ± 2 K using a Bruker Smart CCD area detector with graphite-monochromated Mo Kα radiation (λ = 0.71073 Å). Elemental analysis was performed by the Analytical Center of the University of Science and Technology of China. Molecular weight and molecular weight distribution of the polymer were determined by gel permeation chromatography (GPC) with a PL 210 equipped with one Shodex AT-803S and two Shodex AT-806MS columns at 140 °C using o-dichlorobenzene as a solvent and calibrated with polystyrene standards. DSC measurements were performed on a TA Instruments DSC Q20 machine. Heating rate of 10 °C/min was used and melting temperature (T_m) from the second heating cycle was used. Dichloromethane, toluene, THF, and hexanes were purified by solvent purification systems. Chlorobenzene was distilled from P_2O_5. All other reagents were purchased from commercial sources and used without purification.

Synthesis of anhydrous naphthalene-2-sulfonate lithium compound. A suspension of naphthalene-2-sulfonic acid (4.16 g, 20.0 mmol) in H_2O (100 mL) was stirred at room temperature for 1 h. A solution of p-toluidine (2.14 g, 20.0 mmol) in hydrochloric acid solution (15 mL H_2O with 2 mL concentrated HCl) was added at 0 °C. The reaction mixture was stirred at 0 °C for 4 h. The white precipitate was isolated by filtration, washed with water and dried under vacuum to yield toluidinium naphthalene-2-sulfonate salt at quantitative yield. A suspension of this compound (3.15 g) in THF (200 mL) was cooled to 0 °C. A solution of ^{n}BuLi (4.0 mL, 2.5 M solution in hexane, 10.0 mmol) was added dropwise over 3 min. The mixture was warmed to room temperature over 12 h. The white solid was isolated by filtration, washed with THF (3 × 20 mL) and dried under vacuum to afford a white powder (1.50 g, 70%). The lithium salt was dehydrated using dean-stark apparatus in refluxing toluene.

Standard Procedure for the Synthesis of Ligands L1-L3. L1. ^{n}BuLi (2.8 mL, 7.0 mmol, 2.5 M in hexane) was slowly added to a solution of anhydrous lithium compound (1.50 g, 7.0 mmol) in THF (40 mL) at 0 °C. After stirring for 5 h at room temperature, a solution of chlorodiphenyl phosphine (1.3 mL, 7.0 mmol) in THF (30 mL) was added dropwise and stirred overnight. The volatiles were removed and the residue was taken up in distilled water (80 mL). The mixture was acidified to pH ~2 with concentrated HCl/H_2O solution, stirred for 0.5 h, and extracted several times with CH_2Cl_2 (total volume 100 mL). The extracts were combined, dried over $MgSO_4$, concentrated under vacuum. The crude product was recrystallized from dichloromethane/ether at −20 °C. The resulting white crystals were filtered and dried to give the desired ligand. Yield 39% (1.07 g). ^{1}H NMR (400 MHz, $CDCl_3$) δ 8.66 (d, *J* = 8 Hz, 1H, Ar-*H*), 8.31 (d, *J* = 8 Hz, 1H, Ar-*H*), 8.03 (d, *J* = 7.6 Hz, 1H, Ar-*H*), 7.84–7.59 (m, 4H, Ar-*H*), 7.56–7.41 (m, 8H, Ar-*H*), 7.33 (t, *J* = 2.5, 1H, Ar-*H*). ^{13}C NMR (100 MHz, $CDCl_3$) δ 136.11 (d, *J* = 7.4 Hz, Ar), 135.09 (s, Ar), 134.13 (s, Ar), 134.01 (s, Ar), 132.95 (s, Ar), 130.79 (d, *J* = 5.4 Hz, Ar), 130.27 (s, Ar), 129.68 (s, Ar), 128.42 (s, Ar), 127.90 (s, Ar), 126.34 (s, Ar), 124.43 (s, Ar), 117.02 (s, Ar), 116.20 (s, Ar), 108.51 (s, Ar), 107.35 (s, Ar). ^{31}P NMR (162 MHz, DMSO-D_6) δ -4.93. ESI-MS (m/z): Anal. Calcd. for $C_{22}H_{16}O_3PS$: 391.0558, found: 391.0539 $[M-H]^-$. Anal. Calcd for $C_{22}H_{17}O_3PS$: C, 67.34; H, 4.37; Found: C, 67.39; H, 4.21.

L2. Similar procedures as above were employed except dicyclohexylchlorophosphine (1.62 g, 7.0 mmol) was used. Yield 46% (1.30 g). ^{1}H NMR (400 MHz, $CDCl_3$) δ 9.24 (d, *J* = 8.3 Hz, 1H, Ar-*H*), 8.01

(d, *J* = 4.0, Hz 1H, Ar-*H*), 7.91 (d, *J* = 7.7 Hz, 1H, Ar-*H*), 7.74 (m, 2H, Ar-*H*), 7.53 (m, 1H, Ar-*H*), 5.27 (doft, *J* = 380 Hz, *J* = 6.2 Hz, 1H, P*H*), 3.24 (s, 2H, Cy-*H*), 2.31 (s, 2H, Cy-*H*), 1.97 (d, J = 40.2 Hz, 5H, Cy-*H*), 1.68 (m, 5H, Cy-*H*), 1.57–0.95 (m, 8H, Cy-*H*). ^{13}C NMR (100 MHz, $CDCl_3$) δ 149.31 (s, Ar), 137.52 (d, *J* = 2.0 Hz, Ar), 131.75 (d, *J* = 13.6 Hz, Ar), 130.11 (s, Ar), 129.80 (d, *J* = 8.0 Hz, Ar), 129.08 (s, Ar), 128.41 (s, Ar), 128.03 (s, Ar), 114.49 (s, Ar), 113.44 (s, Ar), 37.68 (s, Cy), 37.23 (s, Cy), 31.22 (s, Cy), 29.32 (d, *J* = 4.1 Hz, Cy), 26.37 (d, *J* = 15.5 Hz, Cy), 25.56 (d, *J* = 1.7 Hz, Cy). ^{31}P NMR (162 MHz, $CDCl_3$) δ 24.17, 15.85. ESI-MS (m/z): Anal. Calcd. for $C_{22}H_{28}O_3PS$: 403.1497, found: 403.1495 [M-H]$^-$. Anal. Calcd. for $C_{22}H_{29}O_3PS$: C, 65.32; H, 7.23; Found: C, 65.37; H, 7.53.

L3. A solution of the anhydrous lithium compound (2.14 g, 10.0 mmol, dehydrated) in THF (40 mL) was cooled to 0 °C and ^{n}BuLi (4.0 mL of a 2.5 M solution in hexanes, 10 mmol) was added dropwise. The yellow mixture was stirred at 0 °C for 30 minutes, cooled to −78 °C, and stirred for another 1.5 h. In a separate flask, THF (30 mL) was cooled to −78 °C and PCl_3 (0.87 mL, 10.0 mmol) was added. The colorless solution was stirred for 15 min. The dilithiated toluene sulfonate solution was cannula transferred to the PCl_3 solution yielding a clear solution. In a third flask, a solution of 2'-bromo-2,6-dimethoxy-1,1'-biphenyl (2.93 g, 10.0 mmol) in THF (40 mL) was cooled to -78 °C, and ^{n}BuLi (4.0 mL of a 2.5 M solution in hexanes, 10.0 mmol) was added dropwise. The resulting pale yellow solution was stirred for 3.5 h and cannula-transferred to the second flask. The yellow solution was stirred for 1 h at -78 °C, allowed to warm to room temperature, and stirred for 2 days. The volatiles were removed under vacuum and the residue was taken up in distilled water (100 mL). The mixture was acidified to pH ~2 with concentrated HCl/H_2O solution, stirred for 0.5 h, and extracted several times with CH_2Cl_2 (total volume 150 mL). The extracts were combined, dried over $MgSO_4$, concentrated under vacuum, and placed in a freezer at −20 °C. Two fractions of a white solid were obtained by filtration. The product was dried under vacuum. Yield 2.48 g (47% based on anhydrous lithium compound). ^{1}H NMR (400 MHz, $CDCl_3$) δ 8.35 (d, *J* = 8.5 Hz, 1H, Ar-*H*), 7.75 (d, *J* = 8.0 Hz, 3H, Ar-*H*), 7.76–7.59 (m, 3H, Ar-*H*), 7.13–6.90 (m, 9H, Ar-*H*), 6.49 (d, J = 8.0 Hz, 1H, Ar-*H*), 6.43 (d, J = 8.0 Hz, 1H, Ar-*H*), 3.63 (s, 3H, Ph-*OMe*), 3.42 (s, 3H, Ph-*OMe*). ^{13}C NMR (100 MHz, $CDCl_3$) δ 159.16 (s, Ar), 150.07 (s, Ar), 135.25 (s, Ar), 134.72 (s, Ar), 134.25 (d, *J* = 5.0 Hz, Ar), 132.47(s, Ar), 131.13 (d, *J* = 13.0 Hz, Ar), 130.80 (s, Ar), 129.86 (s, Ar), 129.44 (s, Ar), 129.26 (s, Ar), 128.48 (s, Ar), 127.71 (s, Ar), 126.97 (d, *J* = 10 .0 Hz, Ar), 126.15 (s, Ar), 125.09 (s, Ar), 124.63 (s, Ar), 122.19 (s, Ar), 120.39 (s, Ar), 111.96 (s, Ar), 110.57 (s, Ar), 110.28 (s, Ar), 109.32 (s, Ar), 108.75 (s, Ar), 107.71 (s, Ar), 54.91 54.74 (s, Ar*OMe*). ^{31}P NMR (162 MHz, $CDCl_3$) δ 1.0. ESI-MS (m/z): Anal. Calcd. for $C_{30}H_{24}O_5PS$: 527.1082, found: 527.1081 [M-H]$^-$. Anal. Calcd. for $C_{30}H_{25}O_5PS$: C, 68.17; H, 4.77; Found: C, 68.29; H, 4.90.

Standard Procedure for the Synthesis of Complexes Pd1-3 and Pd2''. **Complex Pd1**. Ligand **L1** (392 mg, 1.0 mmol) and [(tmeda)Pd(CH_3)$_2$] (253 mg, 1.0 mmol) were dissolved in dioxane (25 mL) and stirred for 4 h at room temperature. The white precipitate was filtered, washed with diethyl ether (10 mL) and dried in vacuum to yield a grey solid, which was dispersed in DMSO (20 mL) at room temperature. The solvent was removed under reduced pressure. This operation was repeated until a clear DMSO solution was obtained. After the removal of DMSO under reduced pressure, the resulting solid was washed with hexane (10 mL), diethyl ether (10 mL), and filtrated. The solid was dried under vacuum to afford **Pd1** as a brown powder (254 mg, 43%). ^{1}H NMR (400 MHz, $CDCl_3$) δ 8.50 (d, *J* = 8.8 Hz, 1H, Ar-*H*), 8.11 (d, *J* = 10.0, 1H, Ar-*H*), 7.83 (d, *J* = 12.0, 1H, Ar-*H*), 7.67–7.56 (m, 6H, Ar-*H*), 7.55–7.32 (m, 6H, Ar-*H*), 7.03 (t, *J* = 9.0 Hz, 1H, Ar-*H*), 2.95 2.41 (s, 6H, DMSO-*H*), 0.50 (s, 3H, Pd-*Me*). ^{13}C NMR (100 MHz, $CDCl_3$) δ151.50 (s, Ar), 136.40 (d, *J* = 1.5 Hz, Ar), 134.01 (s, Ar), 133.89 (s, Ar), 131.35 (d, *J* = 2.2 Hz, Ar), 131.01 (s, Ar), 130.77 (s, Ar), 129.45 (s, Ar), 129.10 (s, Ar), 128.91 (s, Ar), 128.77 (d, *J* = 6.2 Hz, Ar), 128.60 (s, Ar), 128.42 (s, Ar), 128.32 (s, Ar), 128.04 (s, Ar), 40.34 (s, DMSO) 5.89 (s, Pd-*Me*). ^{31}P NMR (162 MHz, DMSO-D_6) δ 18.52. MALDI-TOF (m/z): 574.97 [M-Me]$^+$. Anal. Calcd. for $C_{25}H_{25}O_4PPdS_2$: C, 50.81; H, 4.26; Found: C, 50.69; H, 4.11.

Complex Pd2. Similar procedures as above were employed except **L2** (404 mg, 1.0 mmol) and [(tmeda)Pd(CH_3)$_2$] (253 mg, 1.0 mmol) were used. Yield 41% (247 mg). ^{1}H NMR (400 MHz, $CDCl_3$) δ 9.15 (d, *J* = 8 Hz, 1H, Ar-*H*), 7.93 (d, *J* = 12 Hz, 1H, Ar-*H*), 7.84 (d, *J* = 8 Hz, 1H, Ar-*H*), 7.66–7.58 (m, 3H, Ar-*H*), 3.03 (s, 6H, DMSO-*H*), 2.33 (d, *J* = 8 Hz, 2H, Cy-*H*), 2.05 (s, 2H, Cy-*H*), 1.87–1.69 (m, 10H, Cy-*H*), 1.30–1.17 (m, 8H, Cy-*H*), 0.60 (s, 3H, Pd-*Me*). ^{13}C NMR (100 MHz, $CDCl_3$) δ 148.75 (d, *J* = 6.8 Hz, Ar), 134.34 (d, *J* = 1.9 Hz, Ar), 131.02 (s, Ar), 129.92 (d, *J* = 6.8 Hz, Ar), 129.32 (s, Ar), 128.65 (s, Ar),

127.68 (d, *J* = 5.3 Hz, Ar), 126.19 (s, Ar), 114.56 (s, Ar), 113.25 (s, Ar), 37.80 (s, DMSO), 37.11 (d, *J* = 24 Hz, Cy), 31.03 (d, *J* = 3.6 Hz, Cy), 29.09 (s, Cy), 26.37 (dd, *J* = 15.6 Hz, 5.8 Hz), 25.09 (s, Cy), 2.4 (s, Pd-*Me*). ^{31}P NMR (162 MHz, $CDCl_3$) δ 35.27. MALDI-TOF (m/z): 587.07 [M-Me]$^+$. Anal. Calcd. for $C_{25}H_{37}O_4PPdS_2$: C, 49.79; H, 6.18; Found: C, 49.54; H, 6.05.

Complex Pd3. Similar procedures as above were employed except **L3** (529 mg, 1.0 mmol) and [(tmeda)Pd(CH_3)$_2$] (253 mg, 1.0 mmol) were used. Yield 48% (349 mg). ^{1}H NMR (400 MHz, $CDCl_3$) δ 8.36 (s, 1H, Ar-*H*), 8.17 (d, *J* = 8.0 Hz, 1H, Ar-*H*), 8.07–7.89 (dd, *J* = 11.0, 12.4 Hz, 2H, Ar-*H*), 7.75–7.61 (m, 2H, Ar-*H*), 7.44–7.25 (m, 10H, Ar-*H*), 6.57 (s, 1H, Ar-*H*), 6.15 (s, 1H, Ar-*H*), 3.56 2.59 (s, 6H, OMe-*H*), 2.75 (s, 6H, DMSO-*H*), 0.61 (s, 3H, Pd-*Me*). ^{13}C NMR (100 MHz, $CDCl_3$) δ 156.67 (s, *Ar*OMe), 155.50 (s, Ar), 141.29 (s, Ar), 141.05 (d, *J* = 12.9 Hz, Ar), 136.32 (s, Ar), 135.58 (s, Ar), 134.43 (s, Ar), 133.80 (s, Ar), 132.46 (s, Ar), 131.91 (s, Ar), 131.30 (s, Ar), 130.83 (s, Ar), 129.83 (s, Ar), 129.17 (d, *J* = 5.4 Hz, Ar), 128.56 (s, Ar), 128.15 (s, Ar), 127.30 (s, Ar), 123.52 (s, Ar), 119.96 (s, Ar), 118.43 (s, Ar), 117.67 (d, *J* = 9.4 Hz, Ar), 116.89 (s, Ar), 116.57 (s, Ar), 114.76 (s, Ar), 110.24 (s, Ar), 109.37 (s, Ar), 105.03 (s, Ar), 104.26 (s, Ar), 57.65 57.60 (s, Ar-*OMe*), 54.10 (s, DMSO), 0.05 (s, Pd-*Me*). ^{31}P NMR (162 MHz, $CDCl_3$) δ 2.0. Anal. MALDI-TOF (m/z): 711.03 [M-Me]$^+$. Anal. Calcd. for $C_{33}H_{33}O_6PPdS_2$: C, 54.51; H, 4.57; Found: C, 54.65; H, 4.29.

Complex Pd2''. Similar procedures as above were employed except **[PO⌒Cy]**[1] (354 mg, 1.0 mmol) and [(tmeda)Pd(CH_3)$_2$] (253 mg, 1.0 mmol) were used. Yield 55% (304 mg).^{1}H NMR (400 MHz, $CDCl_3$) δ 8.27 (d, *J* = 8 Hz, 1H, Ar-*H*), 7.61–7.45 (m, 3H, Ar-*H*), 2.30–2.07 (m, 4H, *Cy*), 1.83–1.51 (m, 10H, *Cy*), 1.32–1.09 (m, 8H, *Cy*), 2.95 (s, 6H, DMSO-*H*), 0.66 (s, 3H, Pd-*Me*). ^{31}P NMR (162 MHz, DMSO-D_6) δ 35.41. Anal. MALDI-TOF (m/z): 537.05 [M-Me]$^+$. Anal. Calcd. for $C_{21}H_{35}O_4PPdS_2$: C, 45.61; H, 6.38; Found: C, 45.59; H, 6.21.

Standard Procedure for the Synthesis of Complexes Ni1, Ni2 and Ni3. Complex Ni1. A suspension of Ligand **L1** (392 mg, 1.0 mmol) and Na_2CO_3 (159 mg, 1.75 mmol) in CH_2Cl_2 (25 mL) was stirred at room temperature for 4 hours. *Trans*-[NiCl(Ph)(PPh_3)$_2$] (696 mg, 1.0 mmol) was added to the mixture. The orange colored suspension was stirred at room temperature overnight. After filtration, the solvent was removed under vacuum and the residue was washed with hexane (3 × 20 mL) and Et_2O (3 × 20 mL). After drying under vacuum, complex **Ni1** was obtained as a pure yellow solid. Yield 0.60 g (76%). ^{1}H NMR (400 MHz, $CDCl_3$) δ 8.85 (d, *J* = 8.8 Hz, 1H, Ar-*H*), 8.41 (dd, *J* = 12.0, 8.5 Hz, 2H, Ar-*H*), 8.32–7.85 (m, 13H, Ar-*H*), 7.75–7.22 (m, 17H, Ar-*H*), 6.85 (dd, *J* = 9.0 Hz, 2H, Ar-*H*), δ 6.75 (d, *J* = 10 Hz, 1H, Ar-*H*). ^{13}C NMR (100 MHz, $CDCl_3$) δ155.01 (s, Ar), 139.40 (d, *J* = 1.5 Hz, Ar), 138.45 (d, *J* = 2.5 Hz, Ar), 137.94 (s, Ar), 136.24 (s, Ar), 135.92 (d, *J* = 5.2 Hz, Ar), 135.27 (s, Ar), 133.89 (s, Ar), 133.04 (s, Ar), 132.89 (s, Ar), 131.94 (s, Ar), 131.45 (d, *J* = 2.2 Hz, Ar), 131.24 (s, Ar), 131.01 (s, Ar), 130.79 (d, *J* = 5.4 Hz, Ar), 130.42 (s, Ar), 130.05 (s, Ar), 129.47 (s, Ar), 128.91 (s, Ar), 128.85 (s, Ar), 128.50 (s, Ar), 128.10 (s, Ar), 127.82 (d, *J* = 7.4 Hz, Ar), 127.04 (s, Ar), 126.60 (s, Ar), 126.05 (s, Ar). ^{31}P NMR (162 MHz, $CDCl_3$) δ 20.20, 18.53(d, J = 270 Hz), -7.81, -9.50 (d, J = 270 Hz). MALDI-TOF (m/z): 448.91 [M-PPh_3-Ph]$^+$. Anal. Calcd. for $C_{46}H_{36}O_3P_2SNi$: C, 69.98; H, 4.60; Found: C, 69.81; H, 4.54.

Complex Ni2. Similar procedures as above were employed except **L2** was used**.** Yield 0.27 g (34%). ^{1}H NMR (400 MHz, $CDCl_3$) δ 8.99 (d, *J* = 5 Hz, 1H, Ar-*H*), 8.56 (d, *J* = 8.5 Hz, 2H, Ar-*H*), 8.37–8.10 (m, 5H, Ar-*H*), 8.01–7.15 (m, 15H, Ar-*H*), 6.85 (dd, *J* = 9.0 Hz, 2H, Ar-*H*), δ 6.75 (d, *J* = 10 Hz, 1H, Ar-*H*), 2.35 (s, 2H, Cy-*H*), 2.14 (s, 2H, Cy-*H*), 1.95 (d, *J* = 46.1 Hz, 10H, Cy-*H*), 1.54 (s, 8H, Cy-*H*). ^{13}C NMR (100 MHz, $CDCl_3$) δ152.44 (s, Ar), 139.90 (d, *J* = 6.5 Hz, Ar), 137.01 (s, Ar), 136.24 (s, Ar), 135.92 (s, Ar), 134.04 (s, Ar), 132.89 (d, *J* = 3.8 Hz, Ar), 131.94 (s, Ar), 131.01 (s, Ar), 130.79 (s, Ar), 130.42 (d, *J* = 5.4 Hz, Ar), 129.47 (s, Ar), 129.24 (s, Ar), 129.12 (s, Ar), 128.91 (s, Ar), 128.85 (s, Ar), 128.50 (s, Ar), 128.10 (s, Ar), 127.82 (d, *J* = 7.4 Hz, Ar), 127.04 (s, Ar), 126.60 (s, Ar), 126.20 (s, Ar), 34.25 (d, *J* = 24 Hz, Cy), 31.52 (d, *J* = 3.6 Hz, Cy), 28.47 (s, Cy), 26.11 (dd, *J* = 12.6, 5.8 Hz), 26.10 (s, Cy). ^{31}P NMR (162 MHz, $CDCl_3$) δ 30.41, 28.53(d, J = 304 Hz), 5.82, 3.94 (d, J = 304 Hz). MALDI-TOF (m/z): 461.08 [M-PPh_3-Ph]$^+$. Anal. Calcd. for $C_{46}H_{48}O_3P_2SNi$: C, 68.93; H, 6.04; Found: C, 68.85; H, 6.34.

Complex Ni3. Similar procedures as above were employed except **L3** was used**.** Yield 0.44 g (48%). ^{1}H NMR (400 MHz, $CDCl_3$) δ 8.45 (d, *J* = 8 Hz, 1H, Ar-*H*), 8.29 (d, *J* = 8.5 Hz, 2H, Ar-*H*), 8.12 (m, 5H, Ar), 8.01–7.55 (m, 12H, Ar-*H*), 7.75–6.22 (m, 15H, Ar-*H*), 6.31 (d, *J* = 12.0 Hz, 2H, Ar-*H*), δ 6.75 (d, *J* = 10.0 Hz, 1H, Ar-*H*), 3.78 (s, 3H, OMe-*H*), 3.45 (s, 3H, OMe-*H*). ^{13}C NMR (100 MHz, $CDCl_3$) δ 154.31 (s, Ar), 145.22 (d, *J* = 10 Hz, Ar), 140.55 (s, Ar), 139.41 (s, Ar), 136.32 (s, Ar), 135.55 (s, Ar), 134.83

(d, *J* = 4.4 Hz, Ar), 134.12 (s, Ar), 133.65 (s, Ar), 132.32 (s, Ar), 131.75 (s, Ar), 130.83 (s, Ar), 129.35 (s, Ar), 128.56 (s, Ar), 128.40 (d, *J* = 3.5 Hz, Ar), 128.15 (s, Ar), 127.44 (s, Ar), 126.88 (s, Ar), 126.31 (s, Ar), 125.76 (s, Ar), 124. 23 (s, Ar), 123.52 (s, Ar), 121.38 (s, Ar), 122.14 (d, *J* = 9.4 Hz, Ar), 121.67 (s, Ar), 120.75 (s, Ar), 120.01 (s, Ar), 119.15 (d, *J* = 8.6 Hz, Ar), 118.28 (s, Ar), 117.45 (s, Ar), 116.55 (s, Ar), 115.76 (s, Ar), 115.24 (s, Ar), 114.92 (s, Ar), 113.35 (s, Ar), 112.57 (s, Ar), 111.37 (d, *J* = 4.9 Hz, Ar), 58 11 (s, Ar-*OMe*). ^{31}P NMR (162 MHz, $CDCl_3$) δ 10.74, 8.88 (d, J = 300 Hz), -12.70, -14.56 (d, J = 300 Hz). MALDI-TOF (m/z): 585.04 [M-PPh_3-Ph]$^+$. Anal. Calcd. for $C_{54}H_{44}O_5P_2SNi$: C, 70.07; H, 4.79; Found: C, 69.85; H, 4.64.

2. Spectra Data

2.1. ^{1}H, ^{13}C, ^{31}P of Compound L1-L3, Complexes Pd1, Pd2, Pd3, Pd2''.

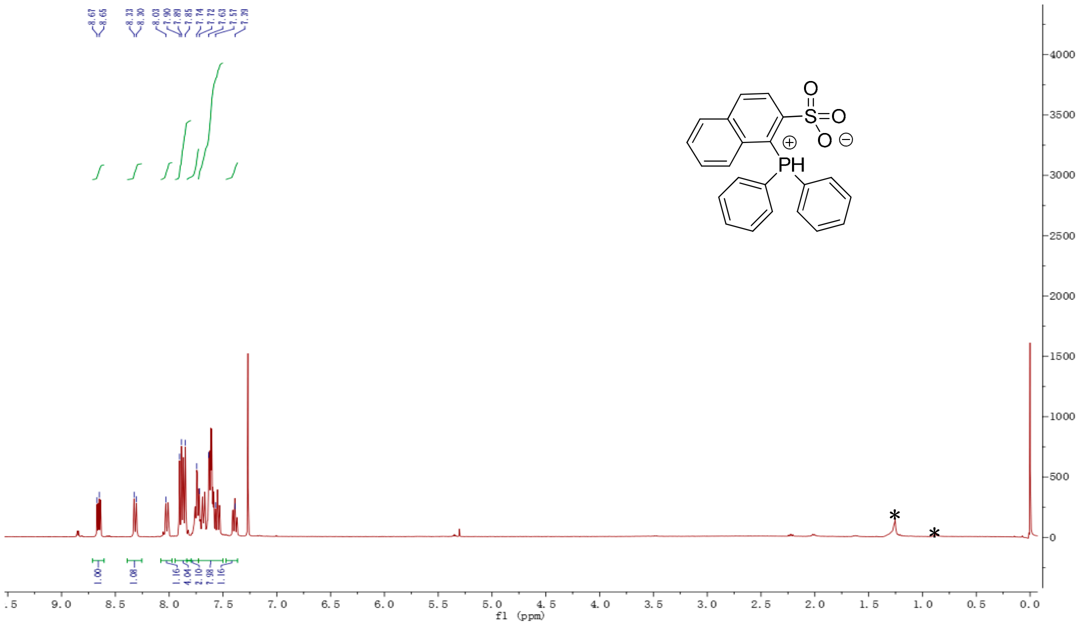

Figure S1. ^{1}H NMR spectrum (400 MHz, $CDCl_3$) of **L1.** * Hexane.

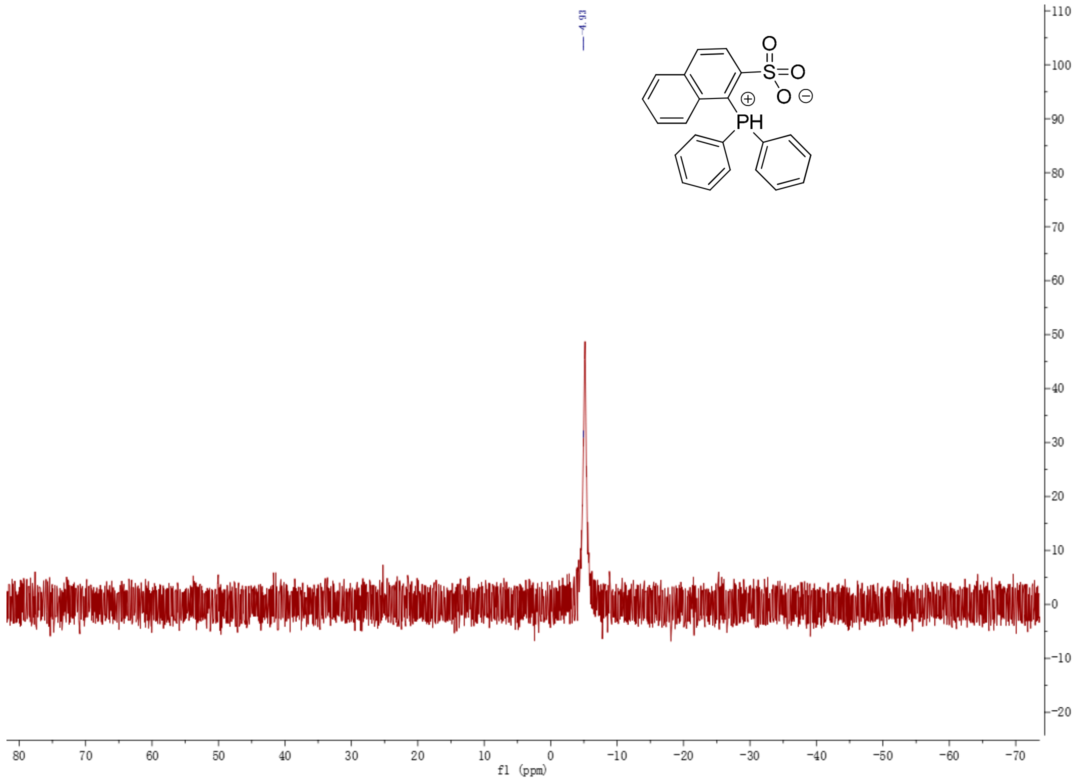

Figure S2. ^{31}P NMR spectrum (162 MHz, $[D_6]DMSO$) of **L1**.

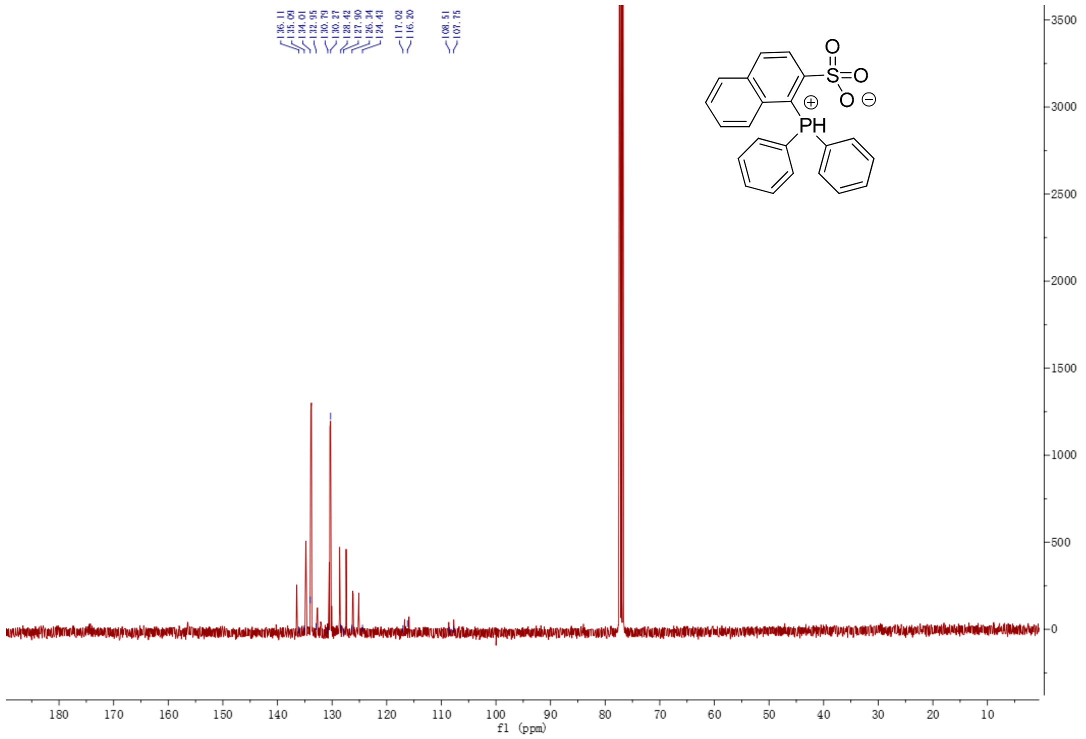

.

Figure S3. ^{13}C NMR spectrum (100 MHz, $CDCl_3$) of **L1.**

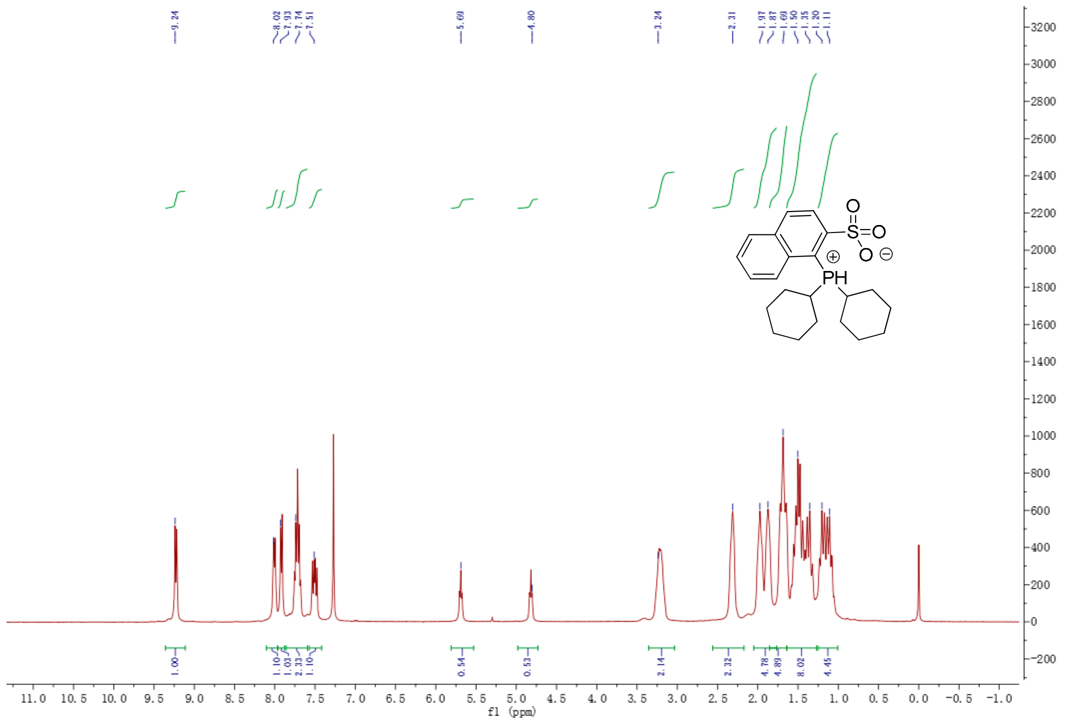

Figure S4. ^{1}H NMR spectrum (400 MHz, $CDCl_3$) of **L2.**

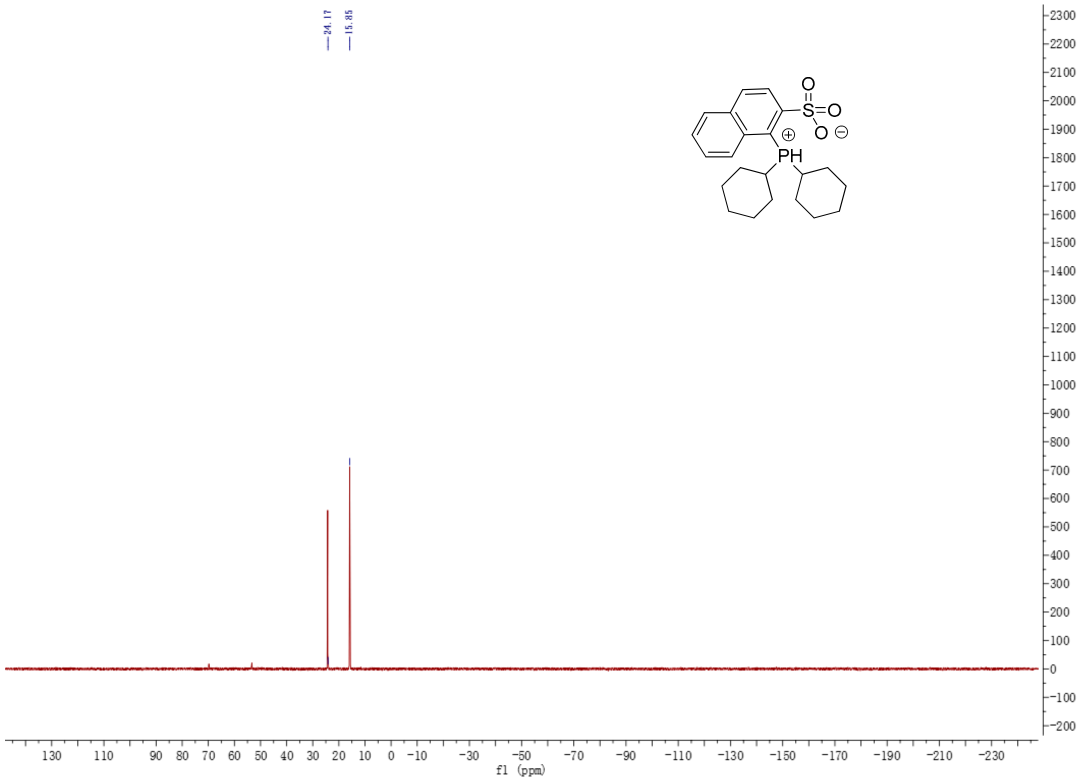

Figure S5. ^{31}P NMR spectrum (162 MHz, $CDCl_3$) of **L2** .

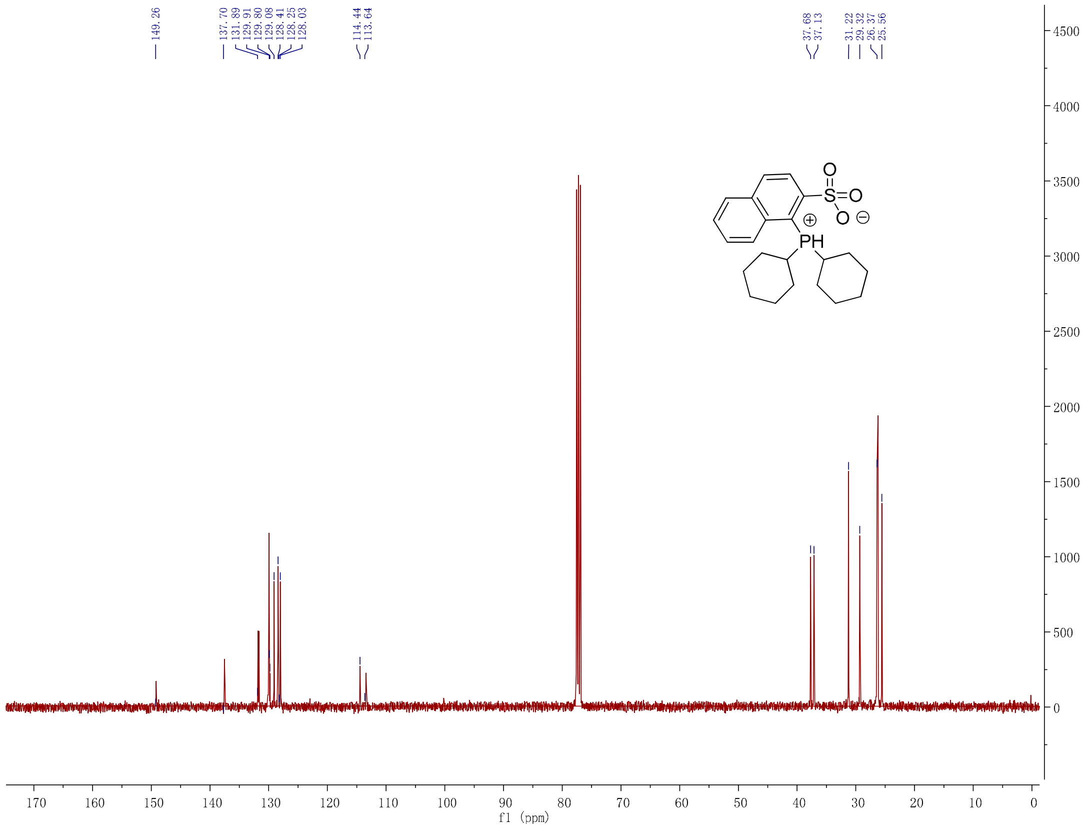

Figure S6. ^{13}C NMR spectrum (100 MHz, $CDCl_3$) of **L2**.

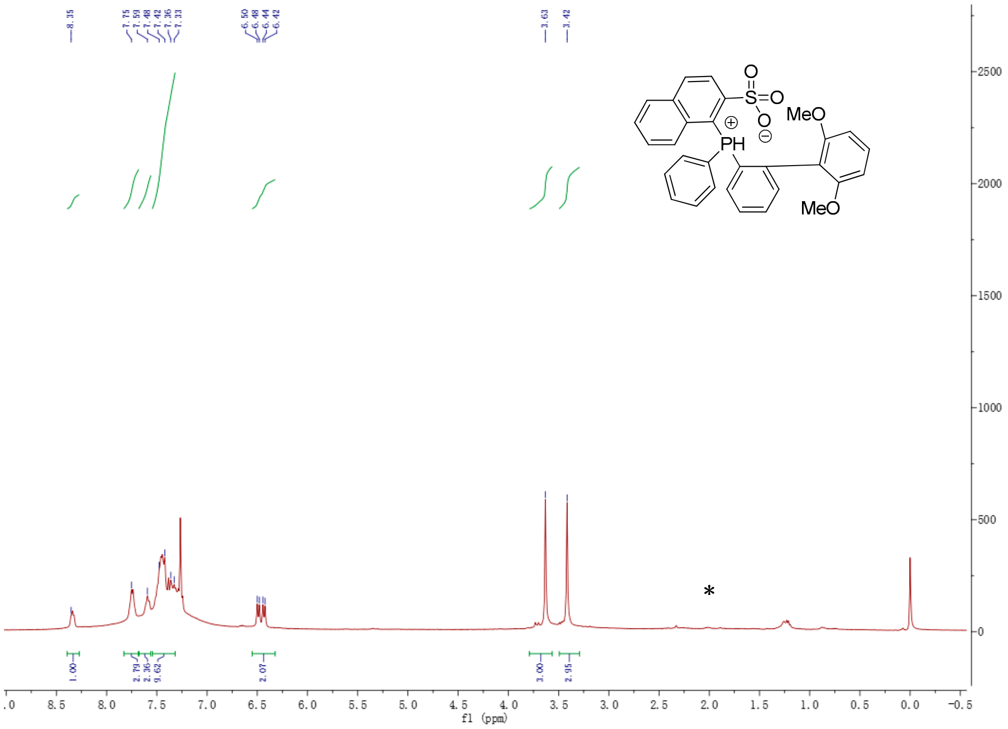

Figure S7. ^{1}H NMR spectrum (400 MHz, $CDCl_3$) of **L3.** * Hexane.

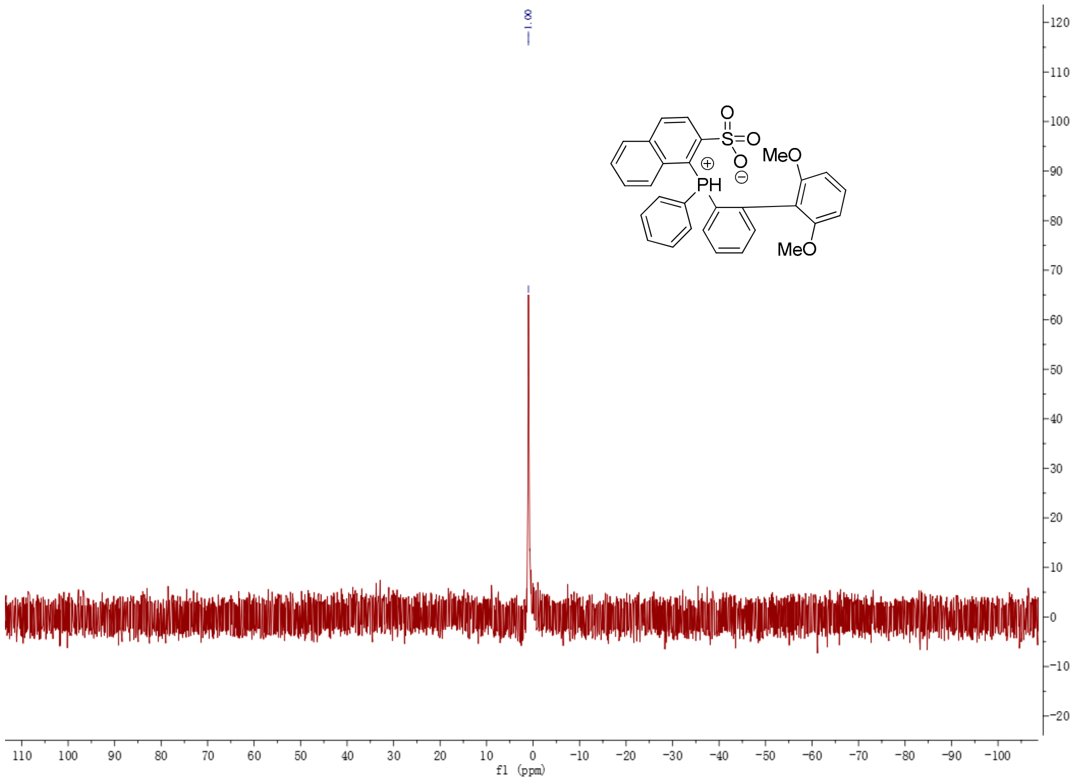

Figure S8. ^{31}P NMR spectrum (162 MHz, $CDCl_3$) of **L3** .

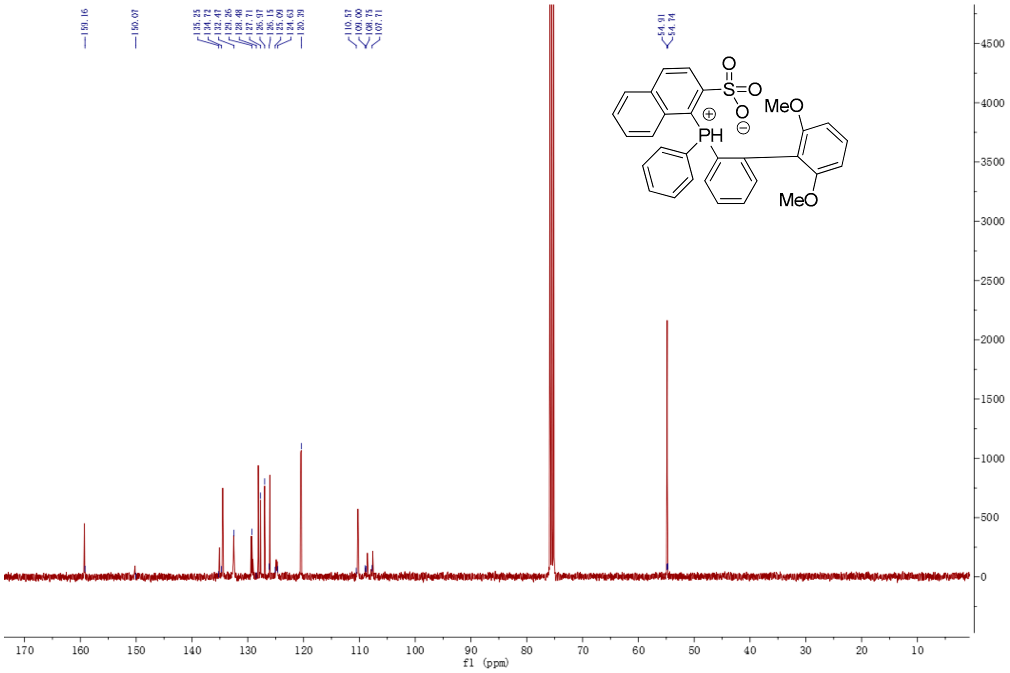

Figure S9. ^{13}C NMR spectrum (100 MHz, $CDCl_3$) of **L3**.

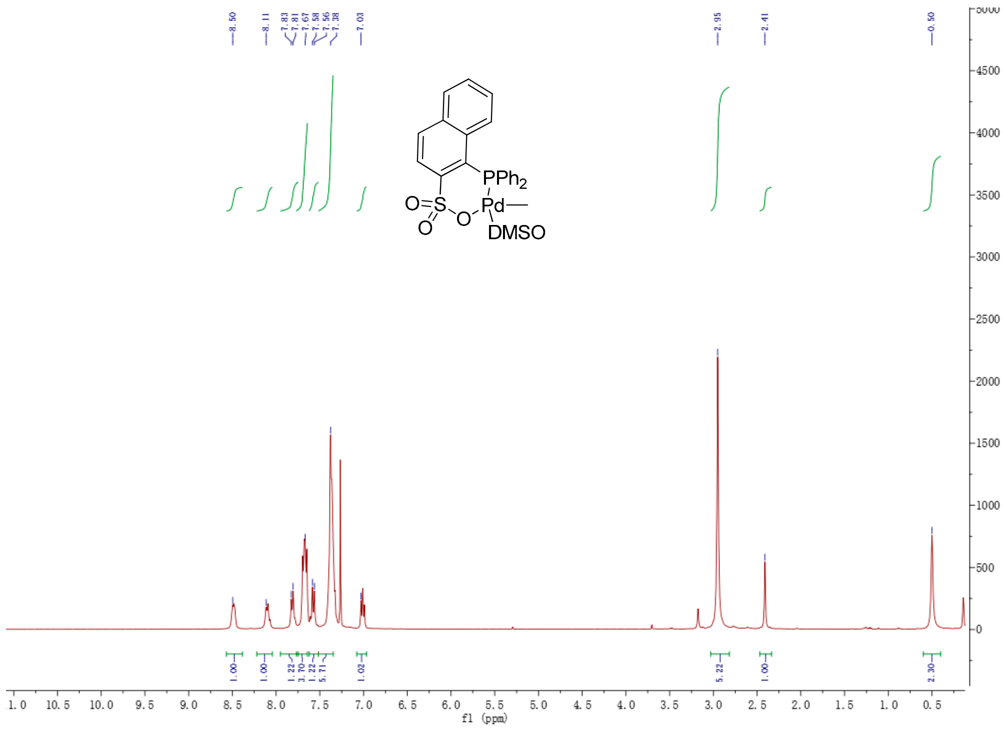

Figure S10. ^{1}H NMR spectrum (400 MHz, $CDCl_3$) of **Pd1.**

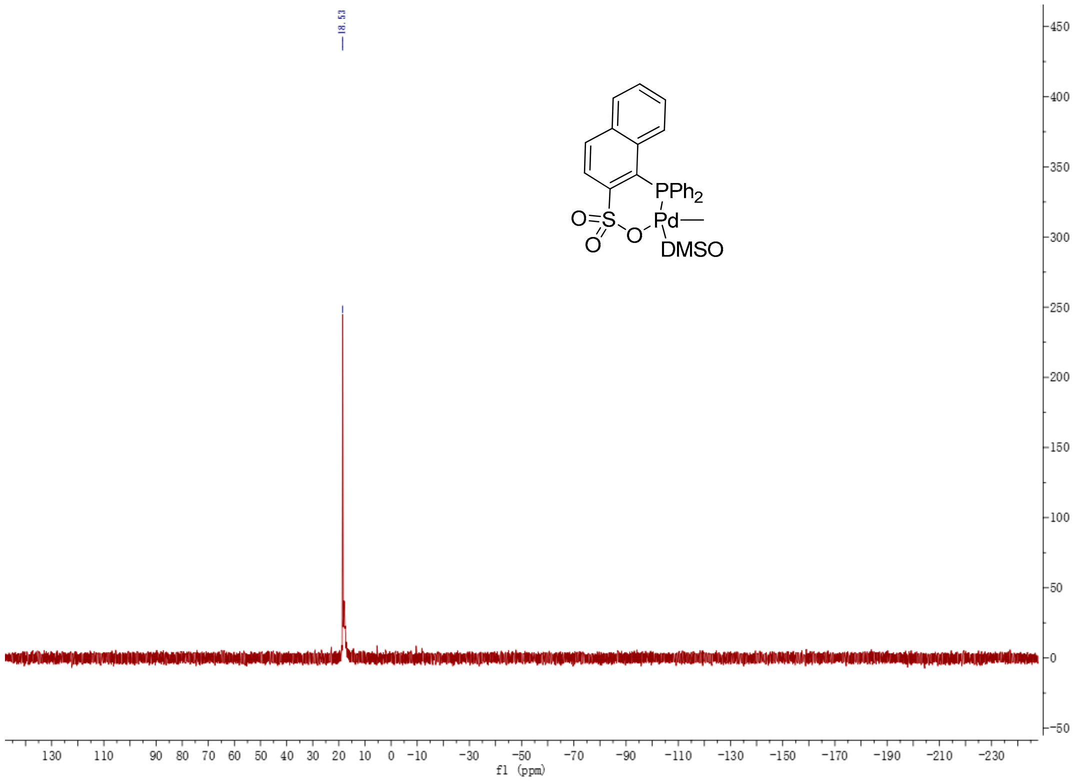

Figure S11. ^{31}P NMR spectrum (162 MHz, $CDCl_3$) of **Pd1**.

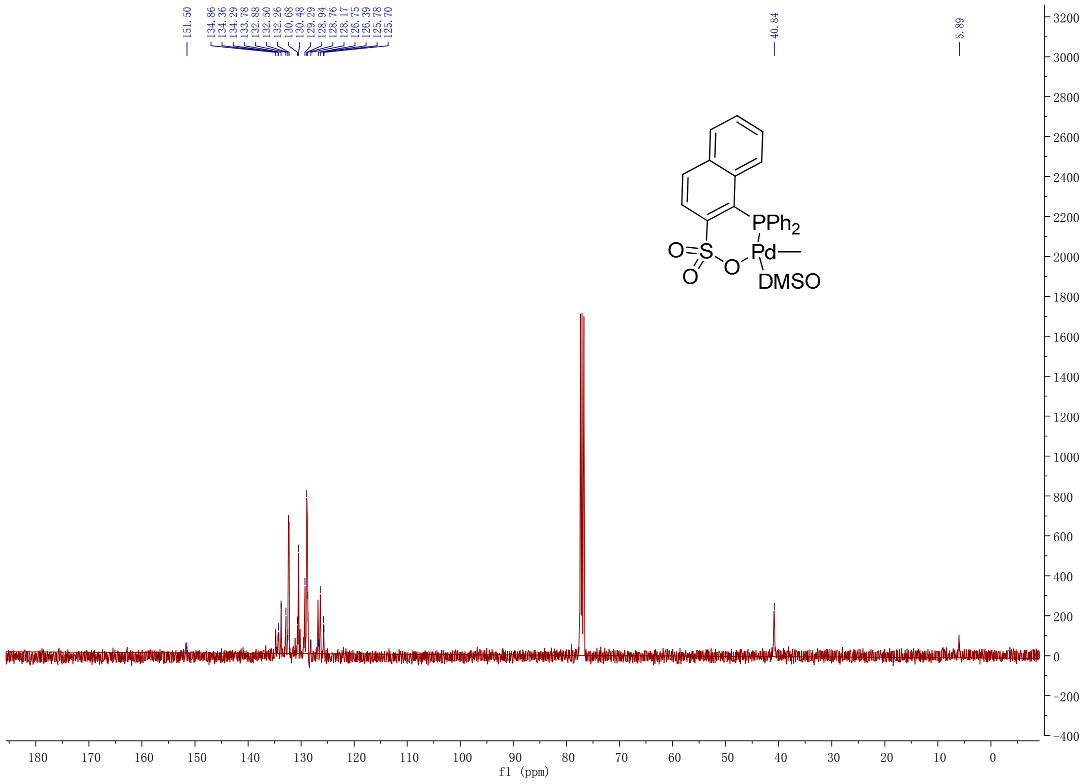

Figure S12. ^{13}C NMR spectrum (100 MHz, $CDCl_3$) of **Pd1**.

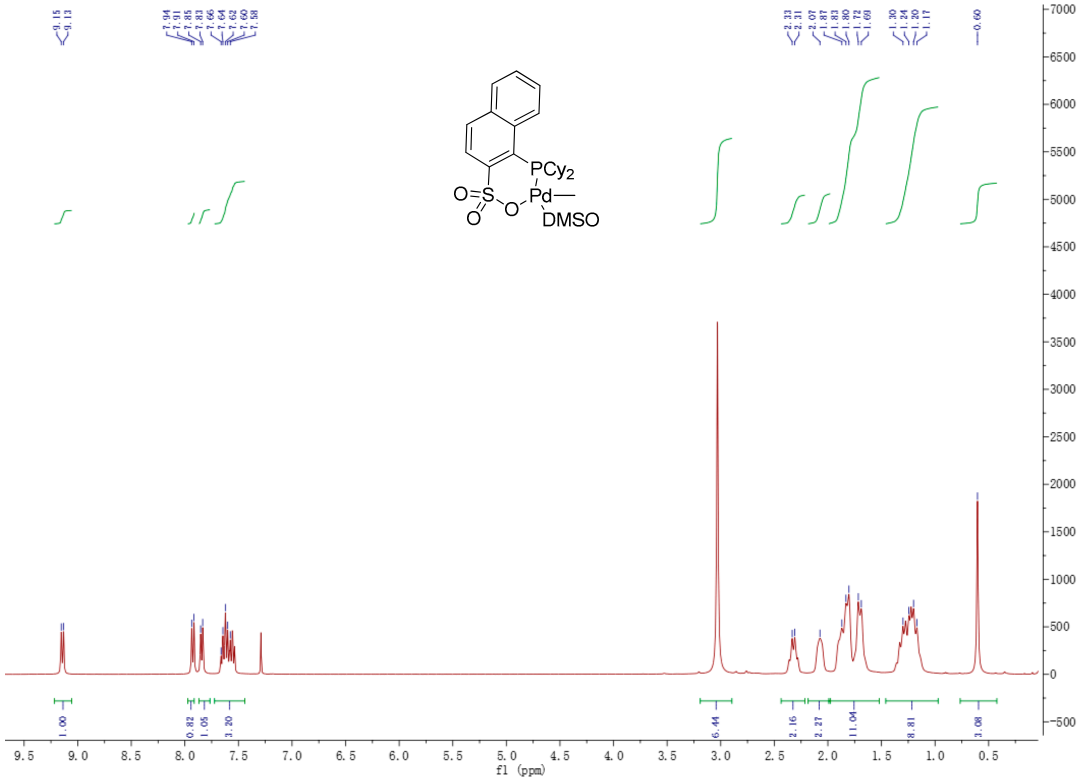

Figure S13. ^{1}H NMR spectrum (400 MHz, $CDCl_3$) of **Pd2.**

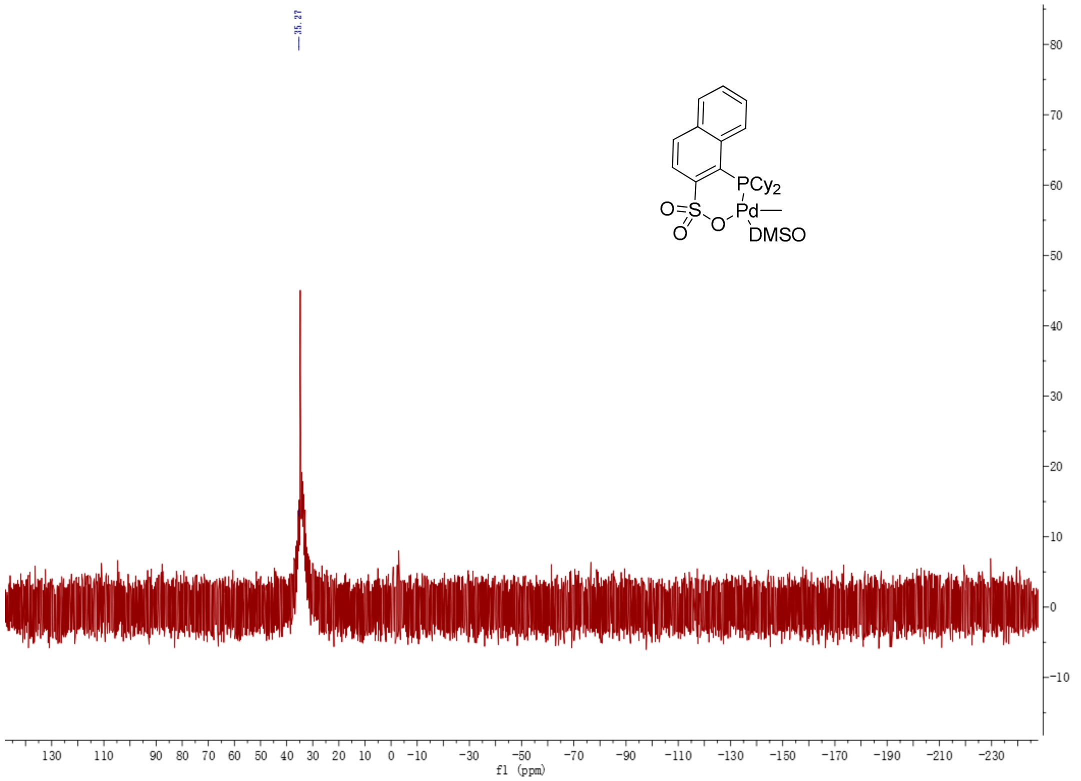

Figure S14. ^{31}P NMR spectrum (162 MHz, $CDCl_3$) of **Pd2**.

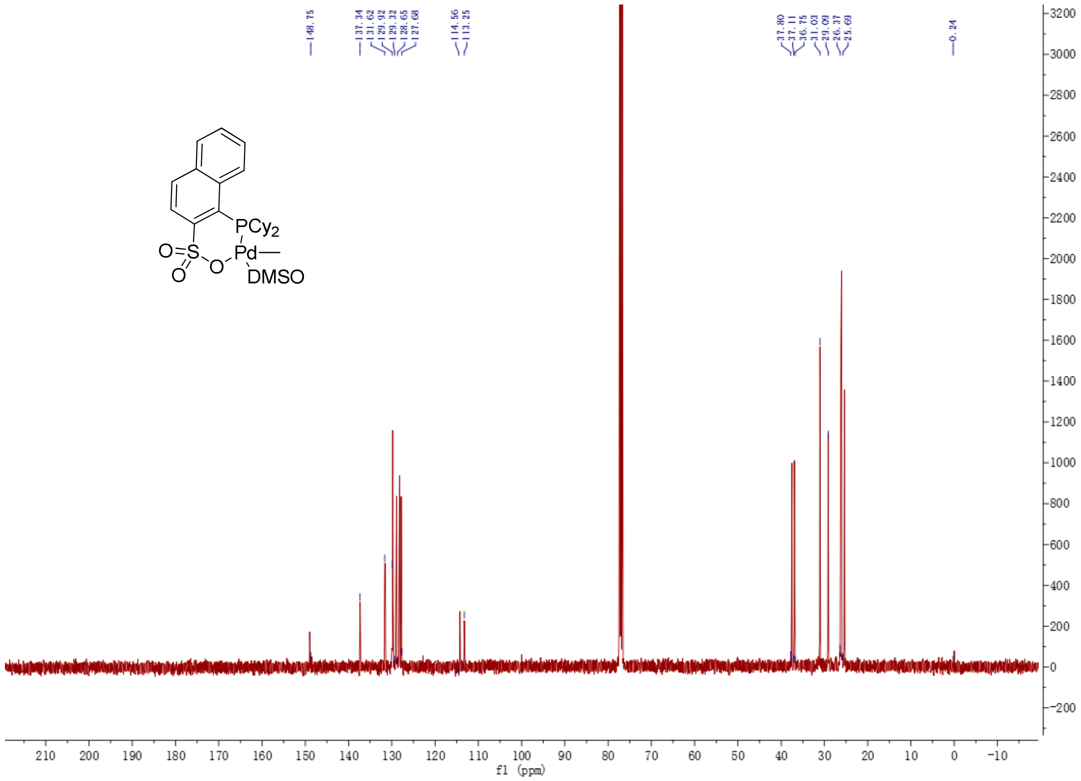

Figure S15. ^{13}C NMR spectrum (100 MHz, $CDCl_3$) of **Pd2**.

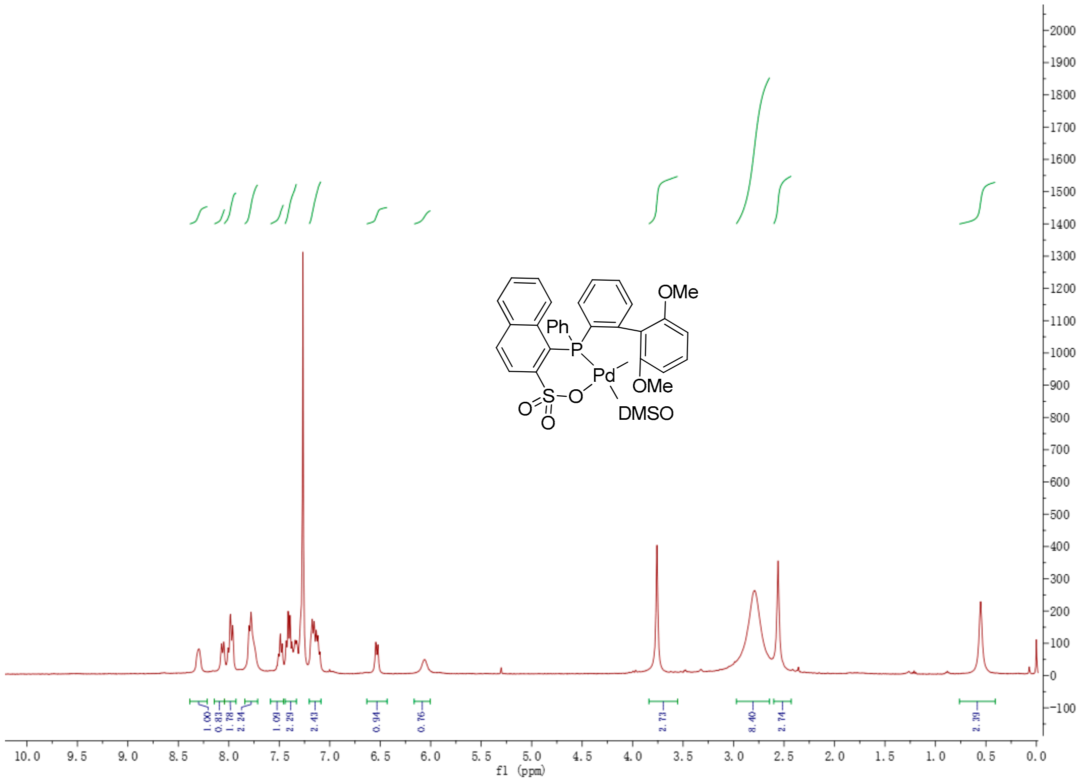

Figure S16. ^{1}H NMR spectrum (400 MHz, $CDCl_3$) of **Pd3.**

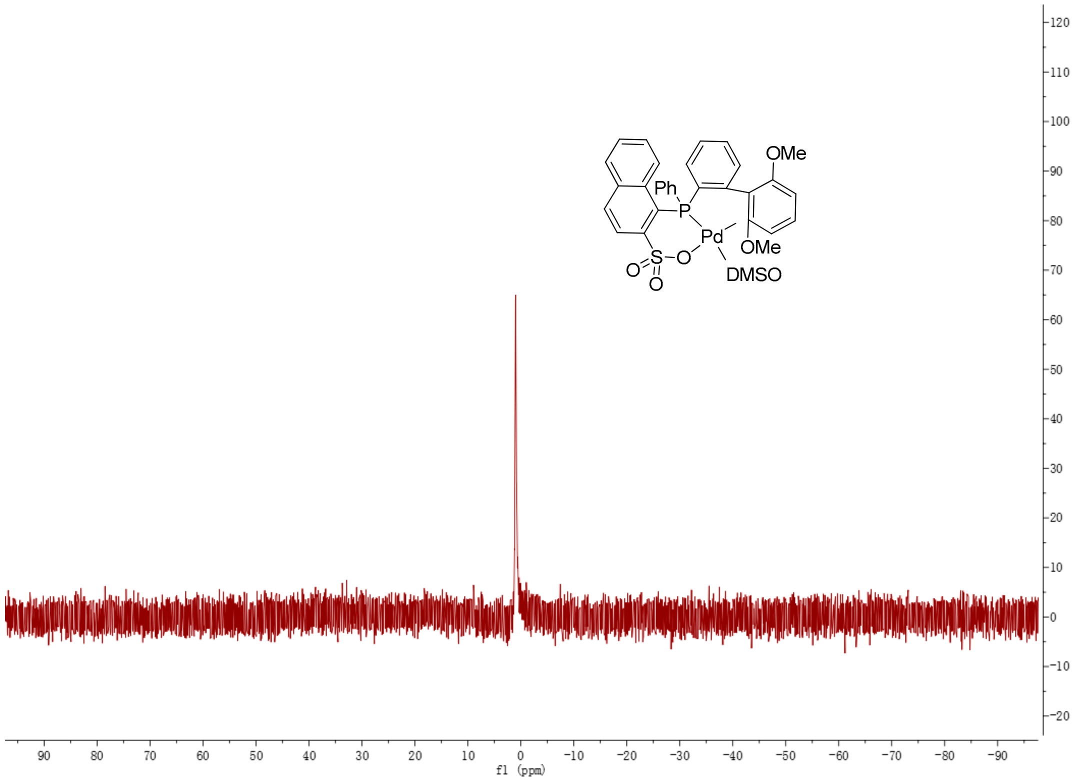

Figure S17. ^{31}P NMR spectrum (162 MHz, $CDCl_3$) of **Pd3**.

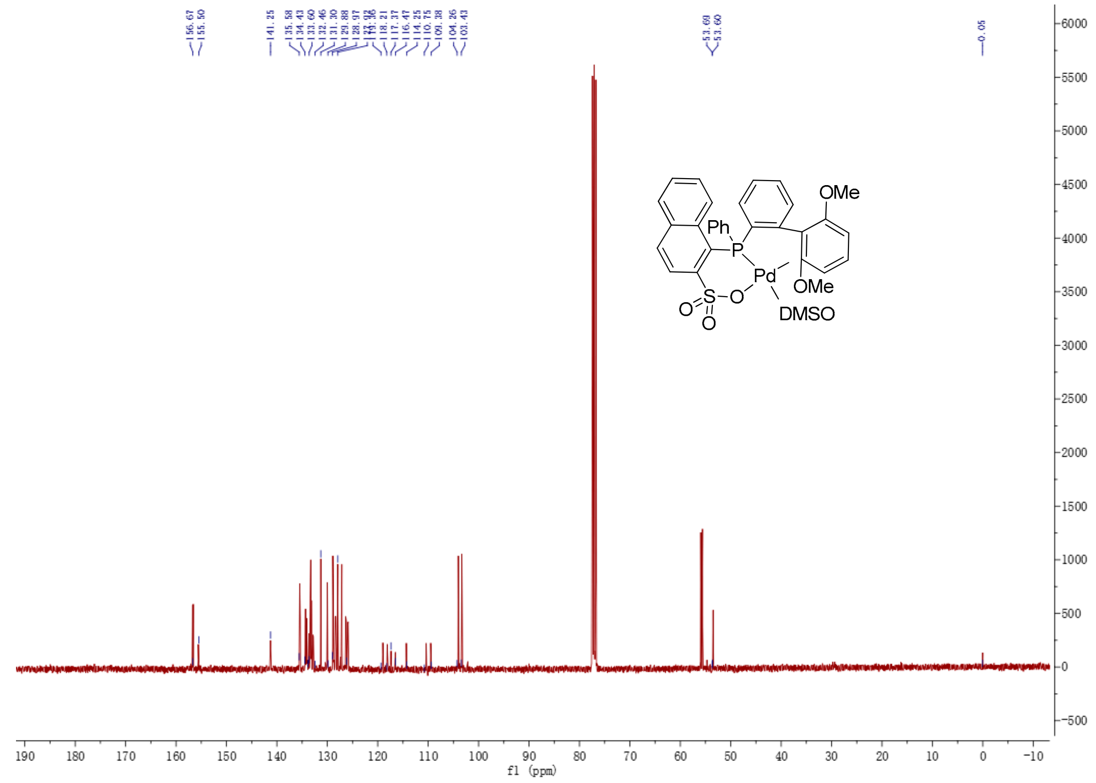

Figure S18. ^{13}C NMR spectrum (100 MHz, $CDCl_3$) of **Pd3**.

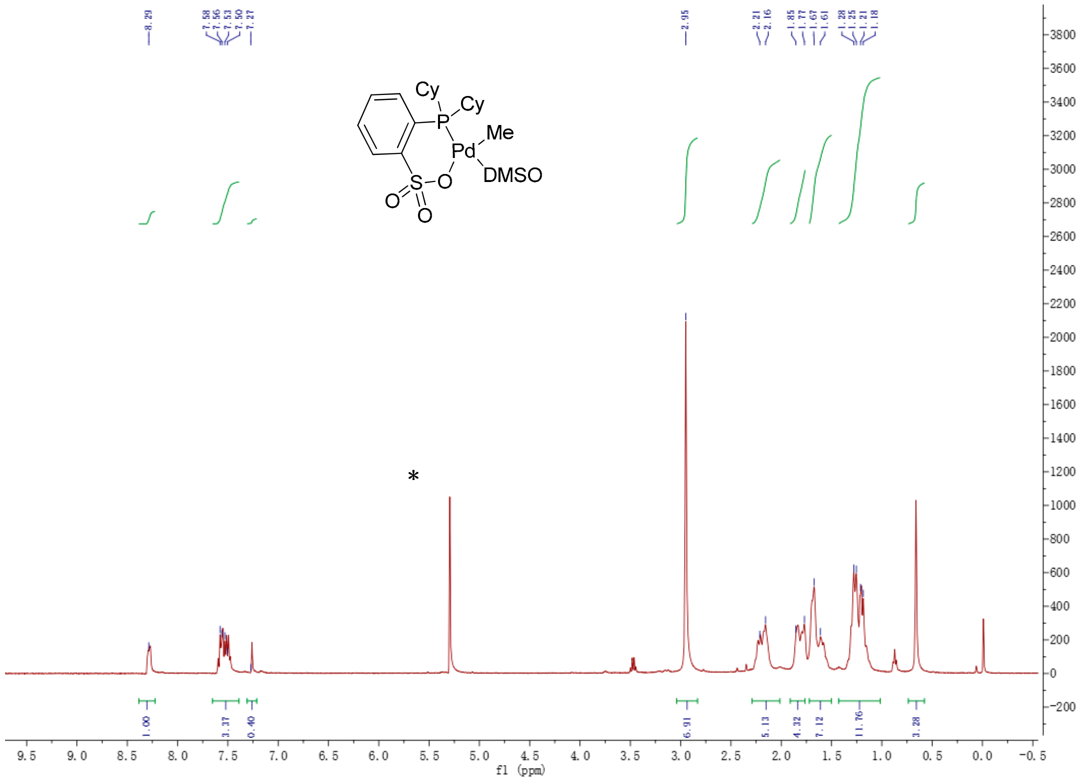

Figure S19. ^{1}H NMR spectrum (400 MHz, $CDCl_3$) of **Pd2''**. * CH_2Cl_2.

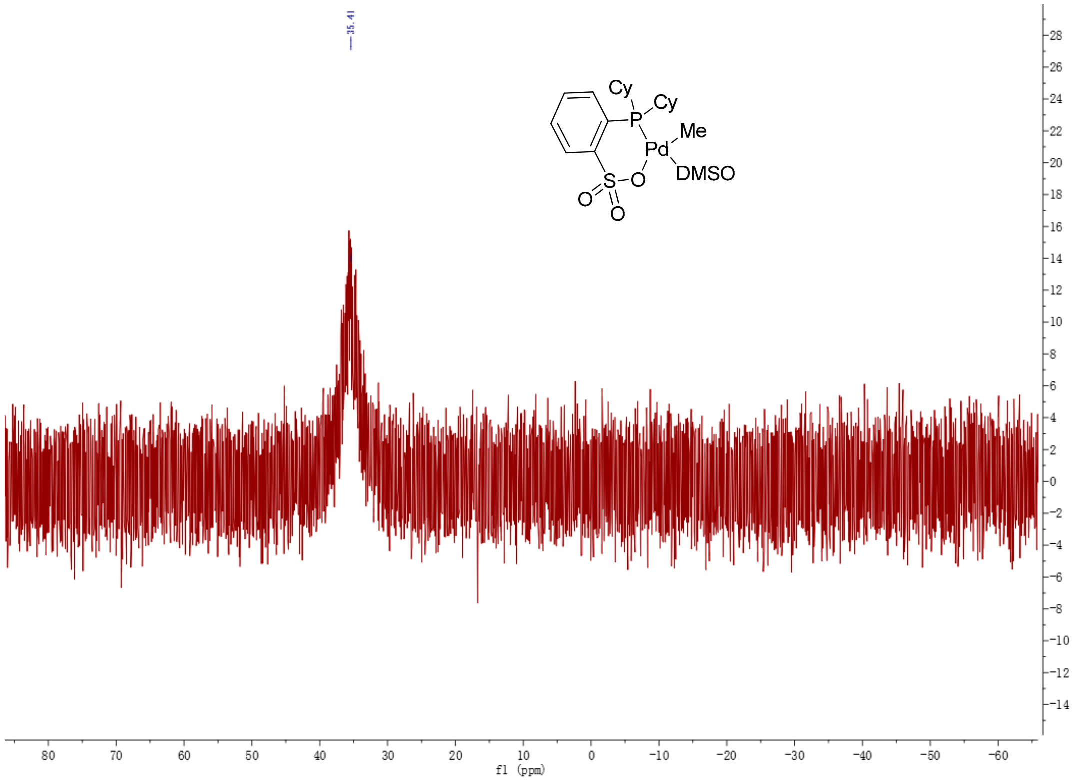

Figure S20. ^{31}P NMR spectrum (162 MHz, $CDCl_3$) of **Pd2''**.

2.2 ^1H of polymer.

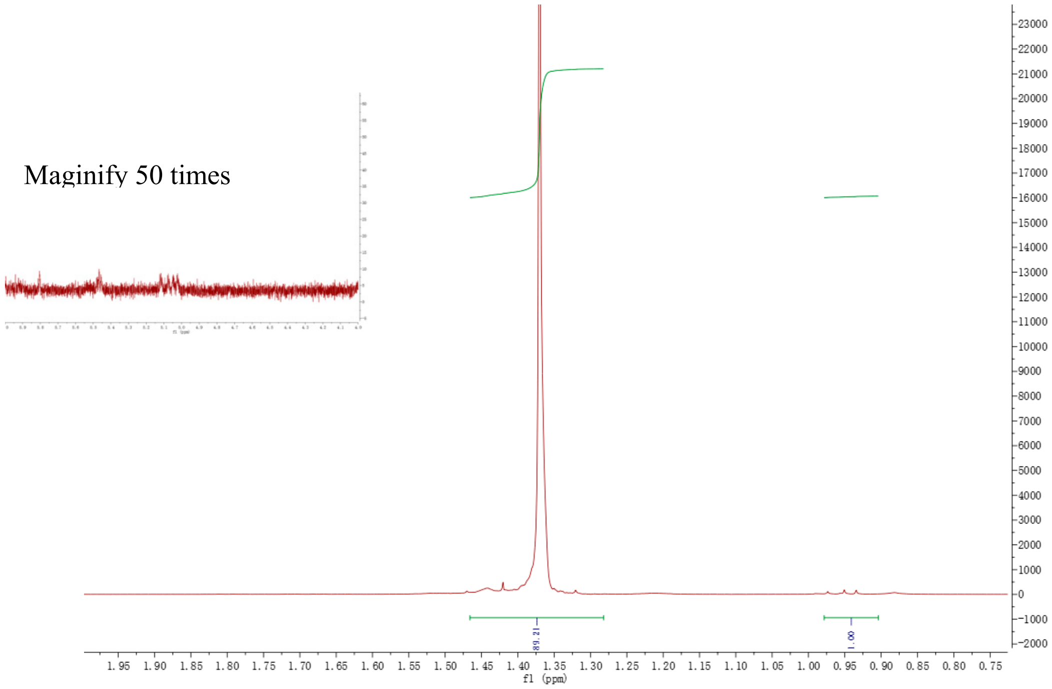

Figure S21. ^1H NMR spectrum of the polymer from Table 1, entry 6 (Insert: the region of δ 5.5-4.0, magnify 50 times). ($C_2D_2Cl_4$, 120 °C).

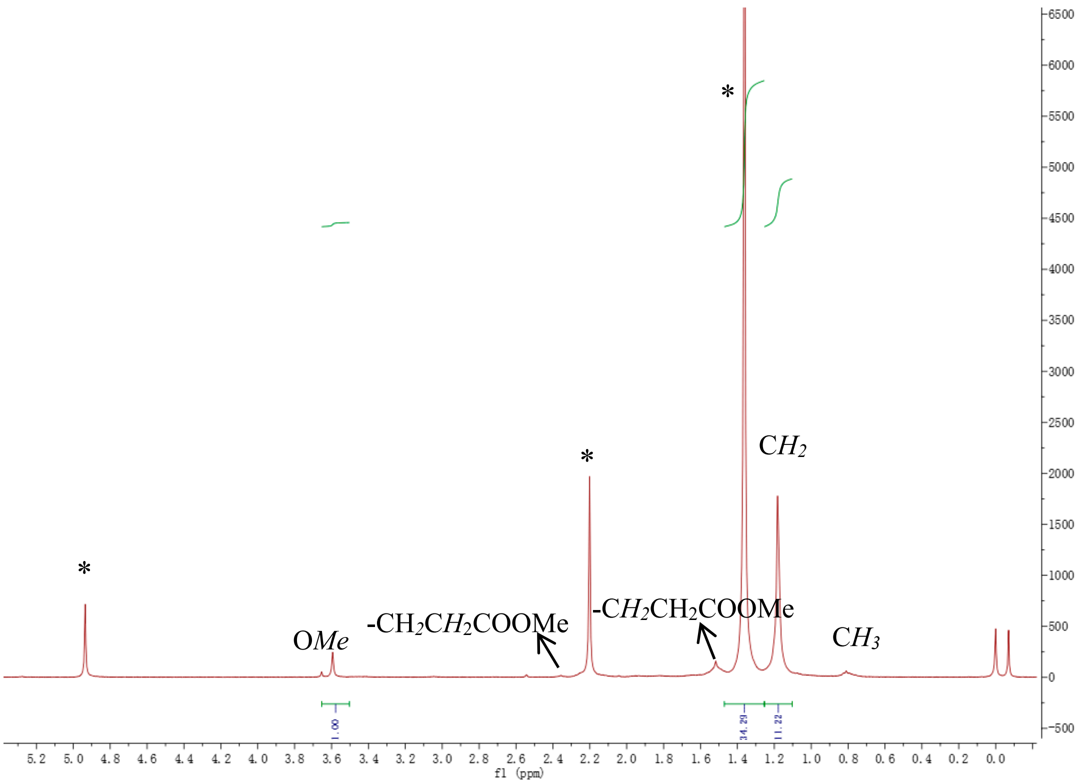

Figure S22. ^{1}H NMR spectrum of the E-MA copolymer generated by complex **Pd3** at 80 °C from Table 2, entry 3 in $CDCl_3$. * BHT.

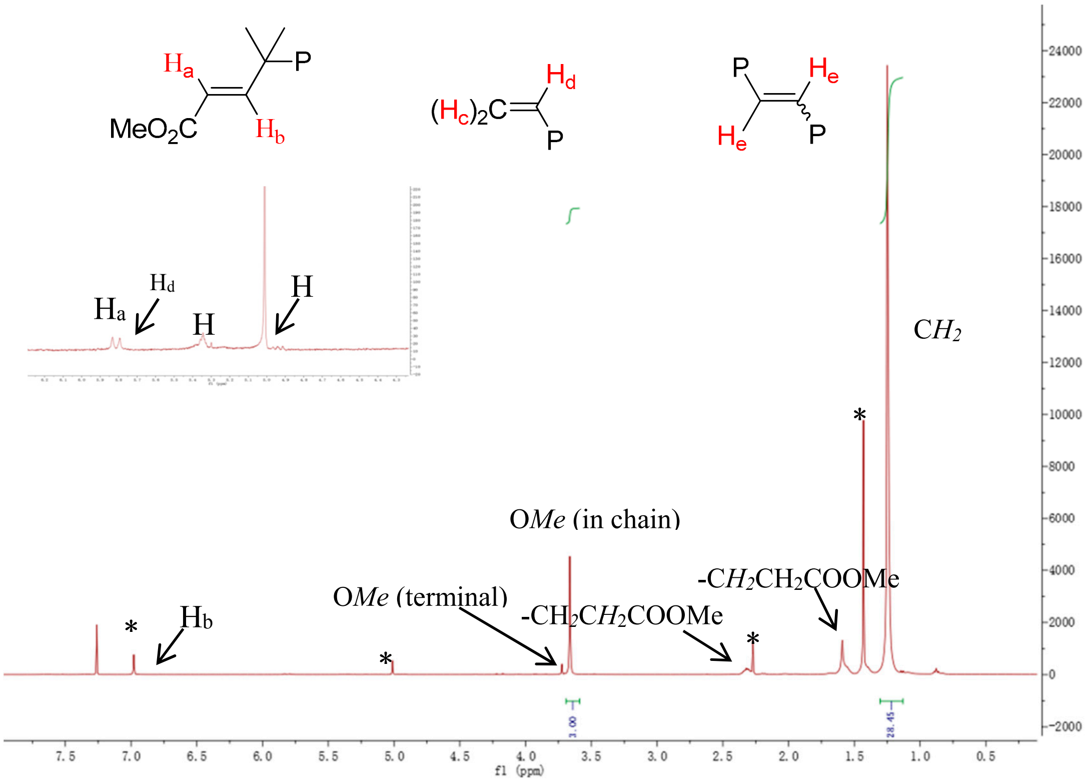

Figure S23. ^{1}H NMR spectrum of the E-MA copolymer generated by complex Pd**2** at 80 °C from Table 2, entry 2 in $CDCl_3$. * BHT. (Insert: the region of δ 6.0-4.0, magnify 50 times).

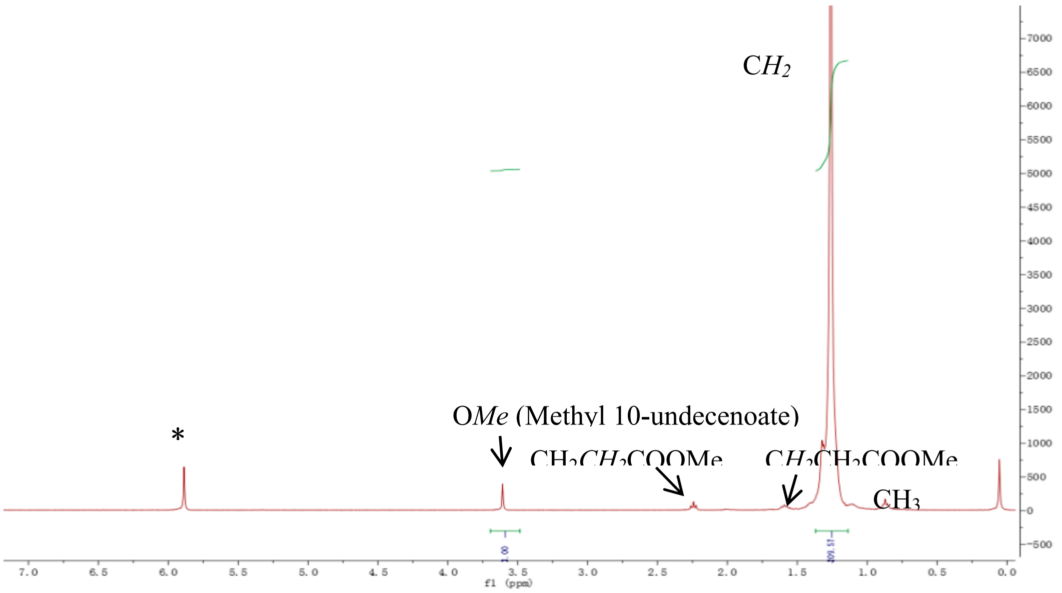

Figure S24. ^{1}H NMR spectrum of the Methyl 10-undecenoate copolymer generated by complex **Ni3** at rt from Table 2, entry 7. * $C_2D_2Cl_4$. ($C_2D_2Cl_4$, 120 °C).

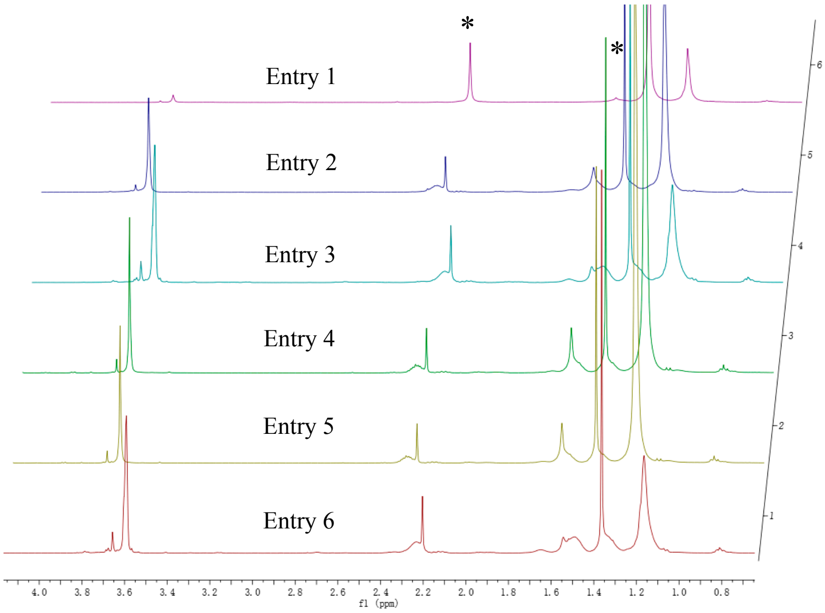

Figure S25. ^{1}H NMR spectrum of the E-MA copolymer from Table 2. * BHT

3. DSC of the polymer.

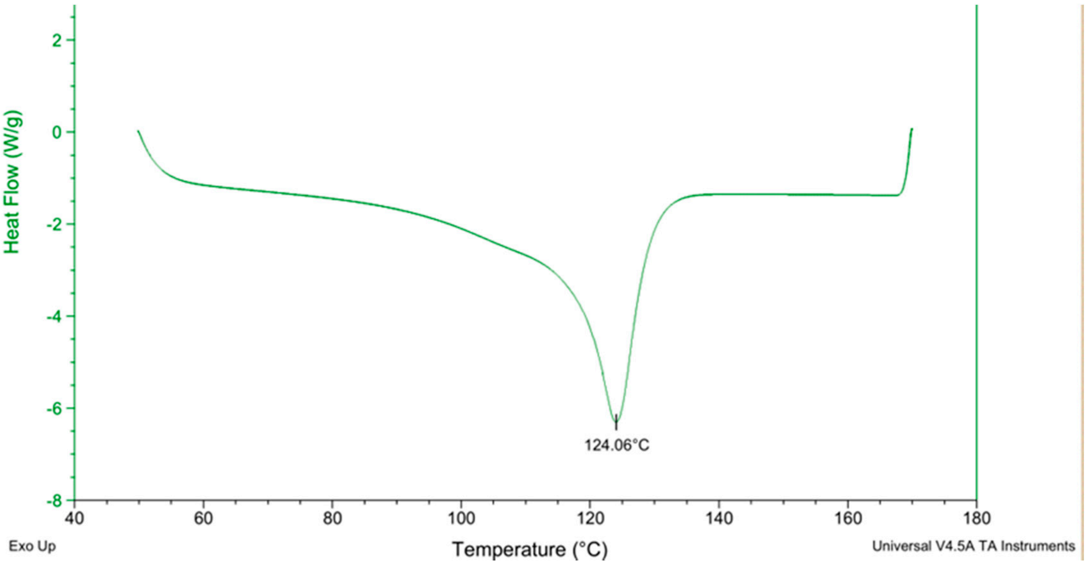

Figure S26. DSC of the polymer from Table 1, entry 5.

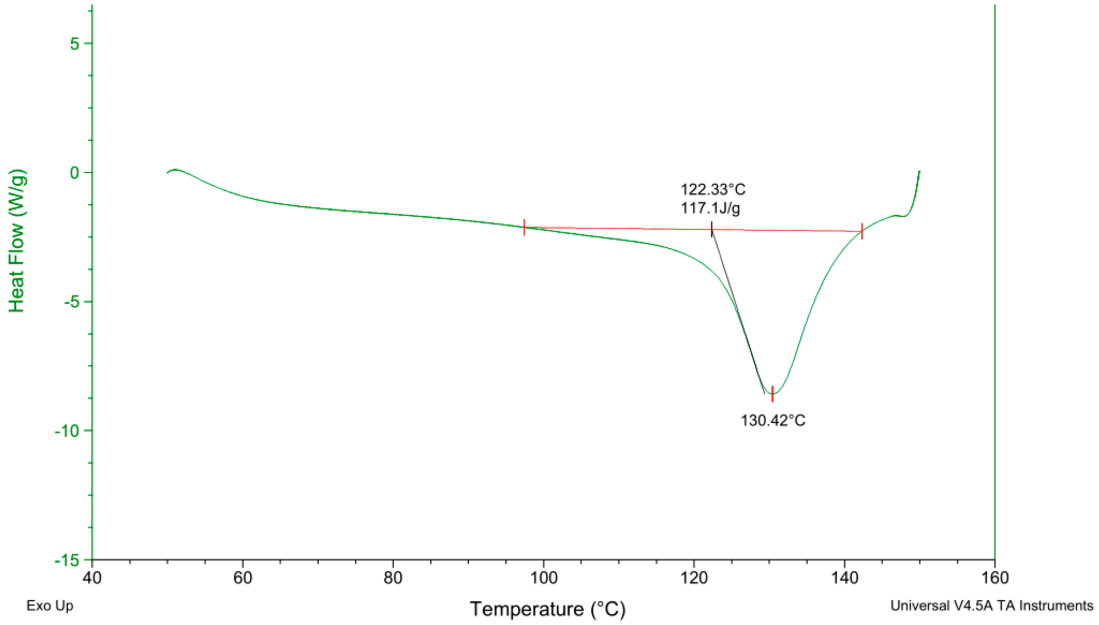

Figure S27. DSC of the polymer from Table 1, entry 6.

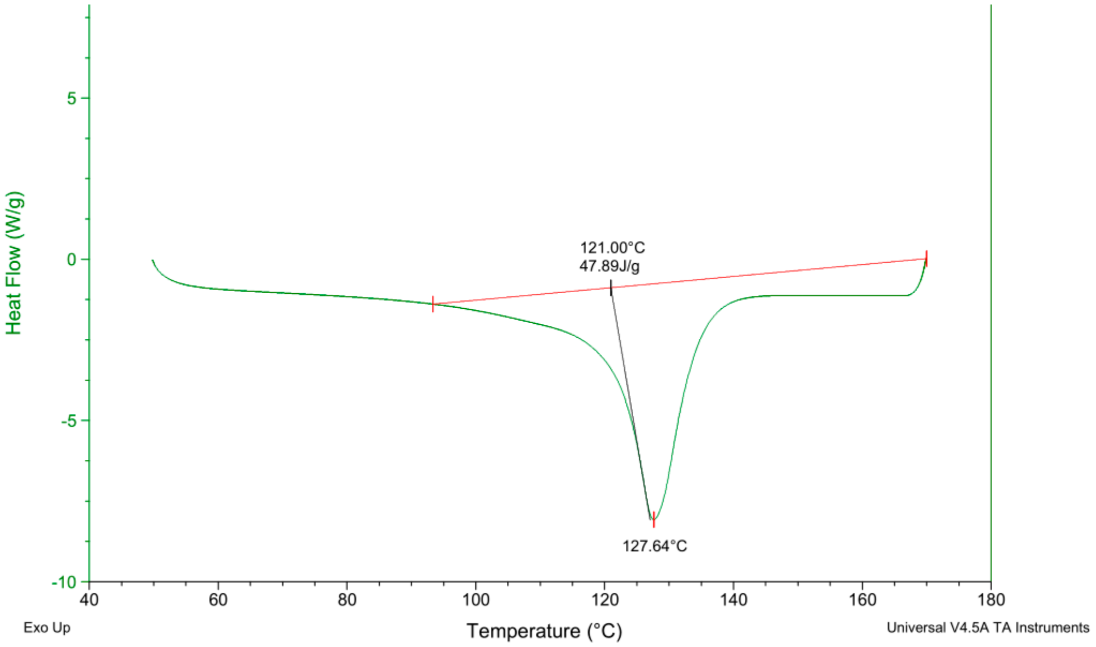

Figure S28. DSC of the polymer from Table 1, entry 7.

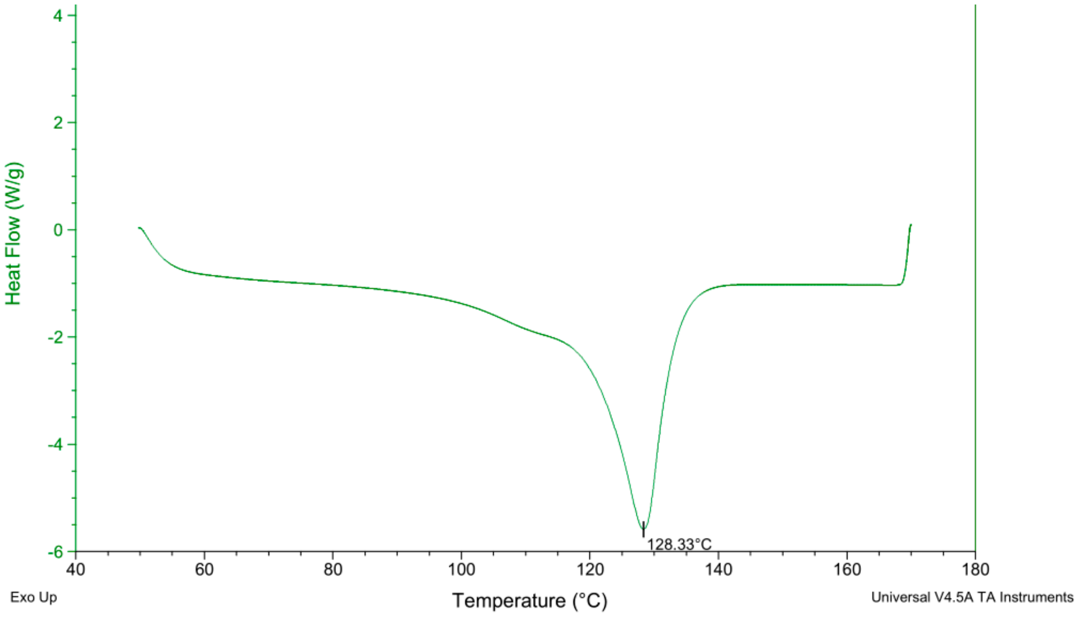

Figure S29. DSC of the polymer from Table 1, entry 8.

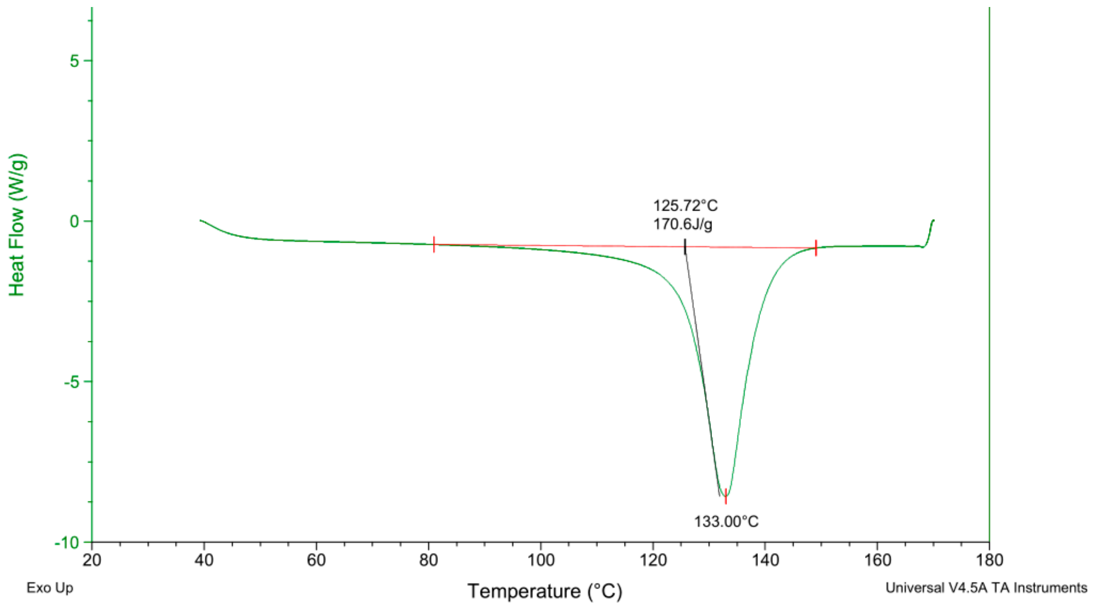

Figure S30. DSC of the polymer from Table 1, entry 9.

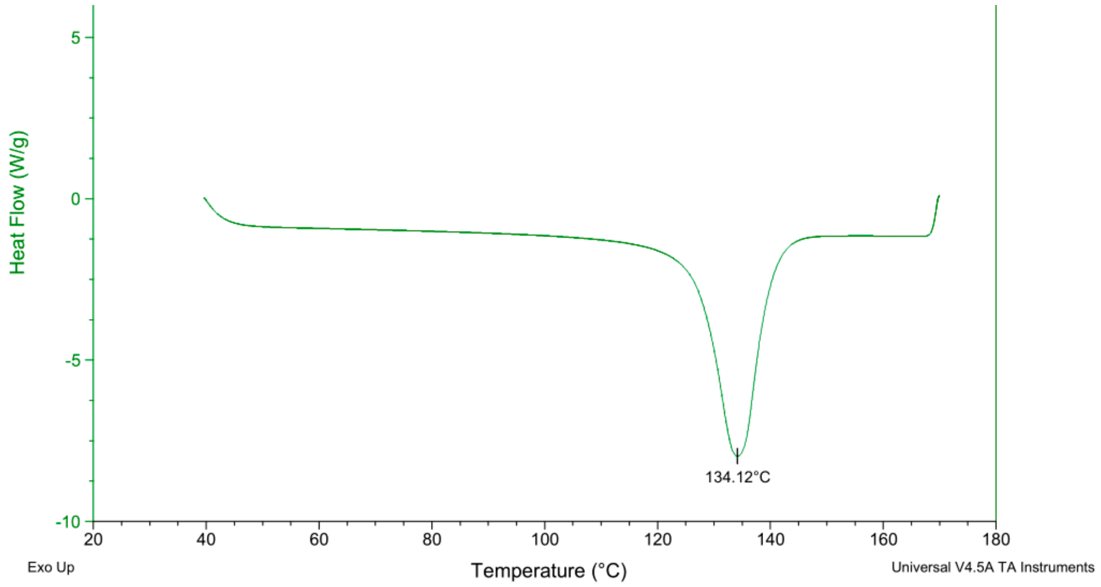

Figure S31. DSC of the polymer from Table 1, entry 10.

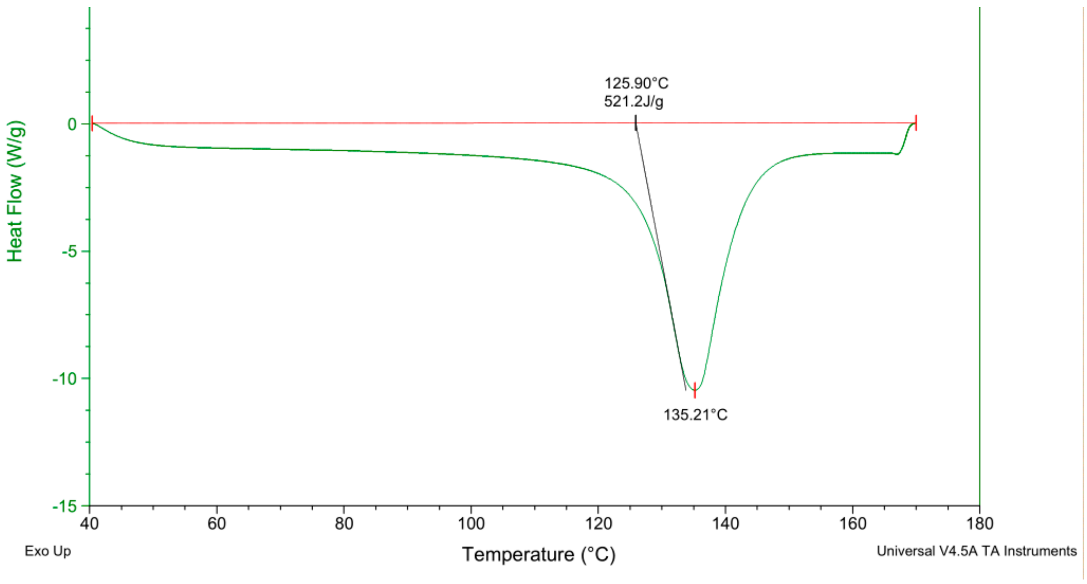

Figure S32. DSC of the polymer from Table 1, entry 11.

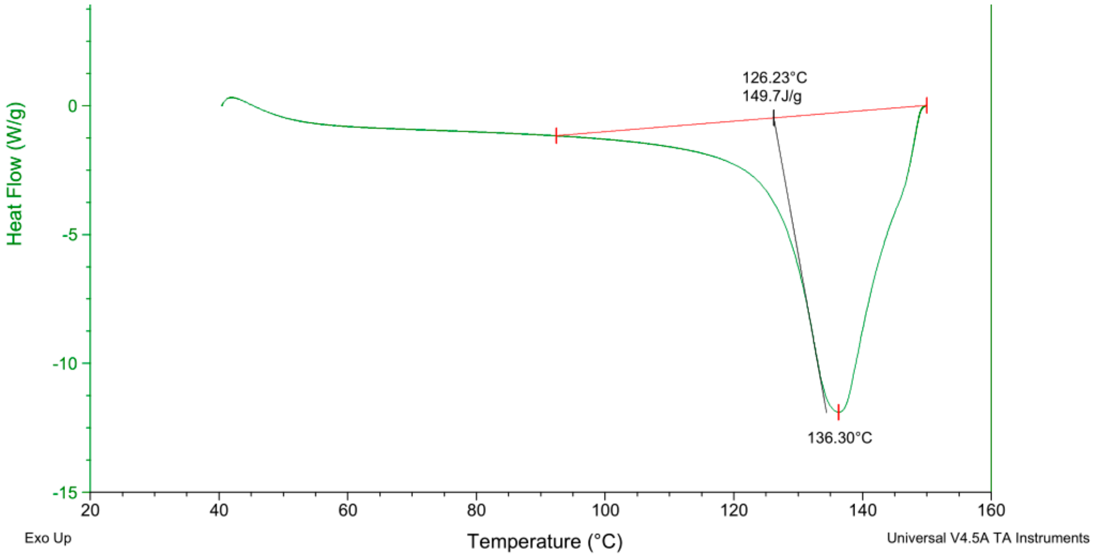

Figure S33. DSC of the polymer from Table 1, entry 12.

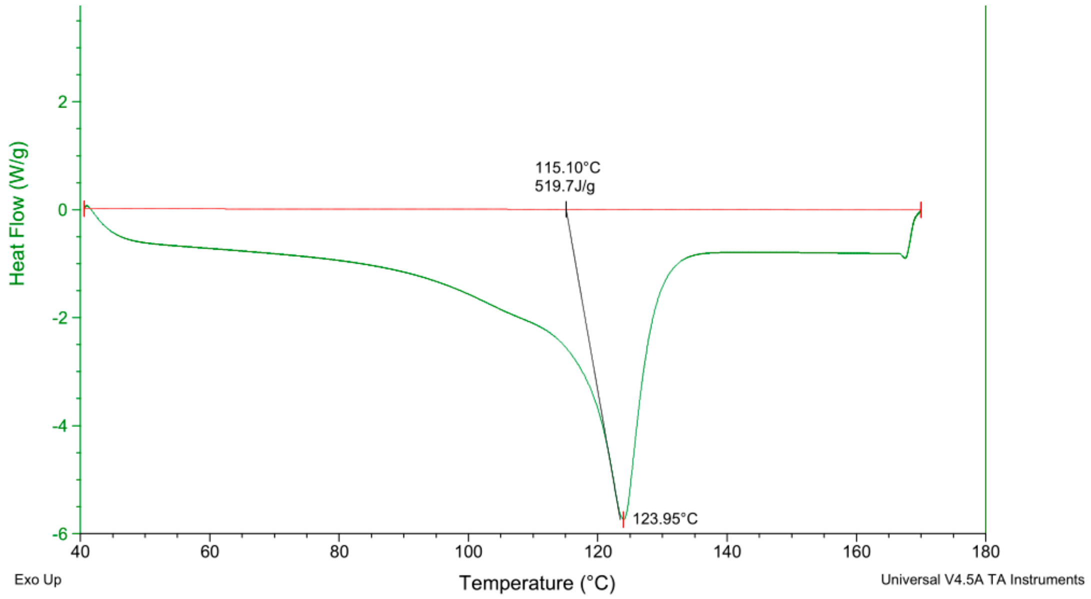

Figure S34. DSC of the polymer from Table 1, entry 13.

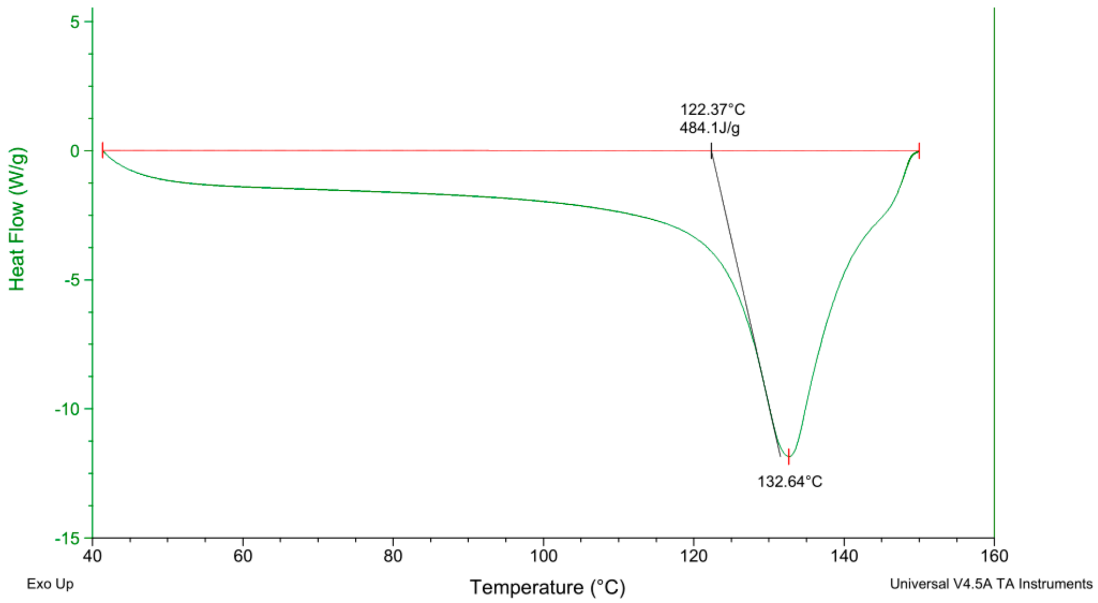

Figure S35. DSC of the polymer from Table 1, entry 16.

4. GPC of the polymer.

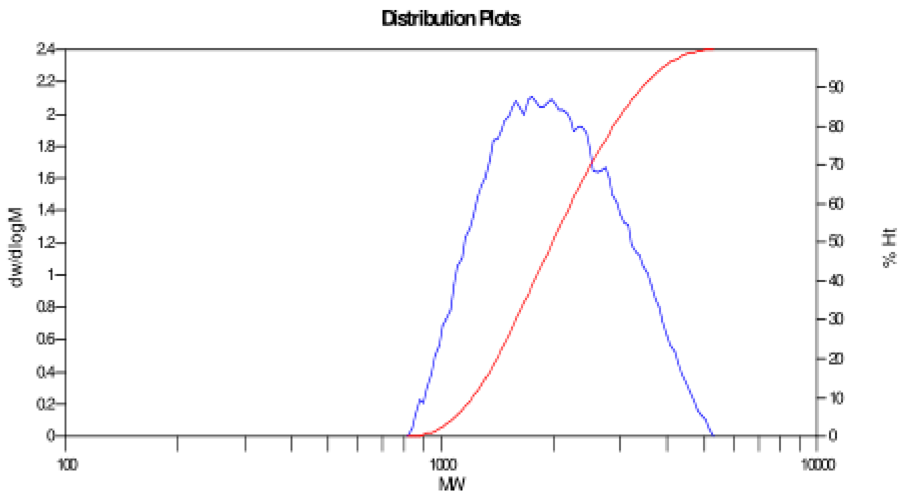

MW Averages

Peak No	Mp	Mn	Mw	Mz	Mz+1	Mv	PD
1	1741	1843	2134	2470	2816	2085	1.15789

Figure S36. GPC of the polymer from Table 1, entry 1.

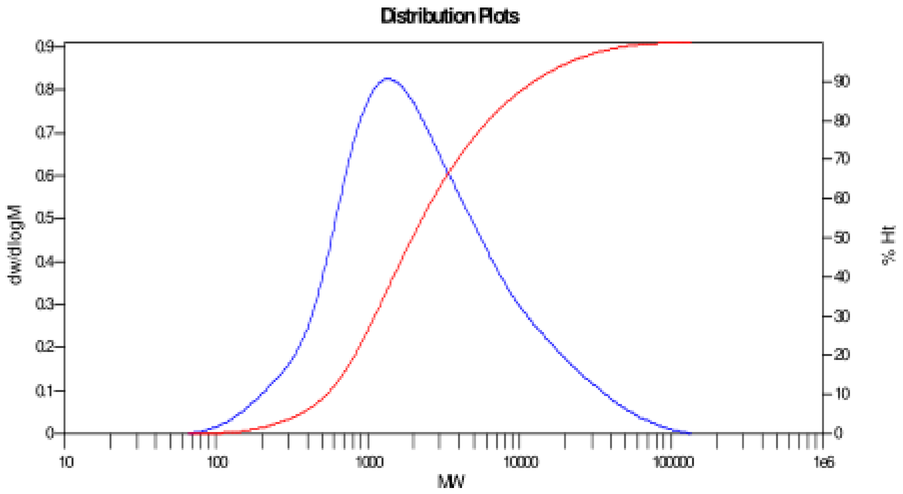

MW Averages

Peak No	Mp	Mn	Mw	Mz	Mz+1	Mv	PD
1	1386	1166	5108	22511	47332	3945	4.38079

Figure S37. GPC of the polymer from Table 1, entry 2.

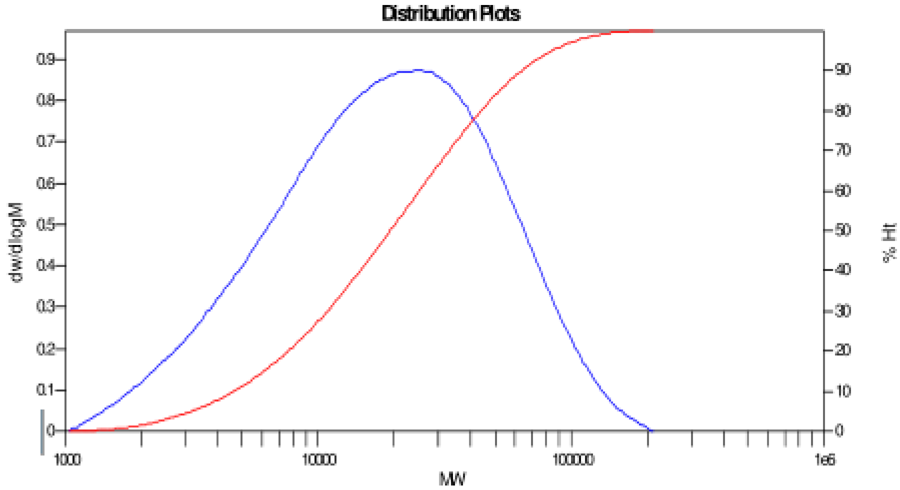

MW Averages

Peak No	Mp	Mn	Mw	Mz	Mz+1	Mv	PD
1	24019	10819	27786	53599	79998	24550	2.56826

Figure S38. GPC of the polymer from Table 1, entry 3.

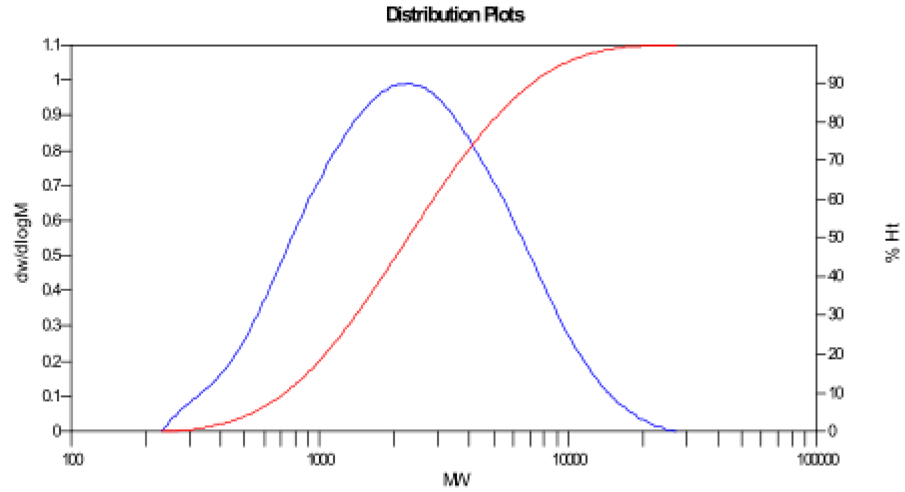

MW Averages

Peak No	Mp	Mn	Mw	Mz	Mz+1	Mv	PD
1	2262	1564	3223	6032	9332	2901	2.06074

Figure S39. GPC of the polymer from Table 1, entry 4.

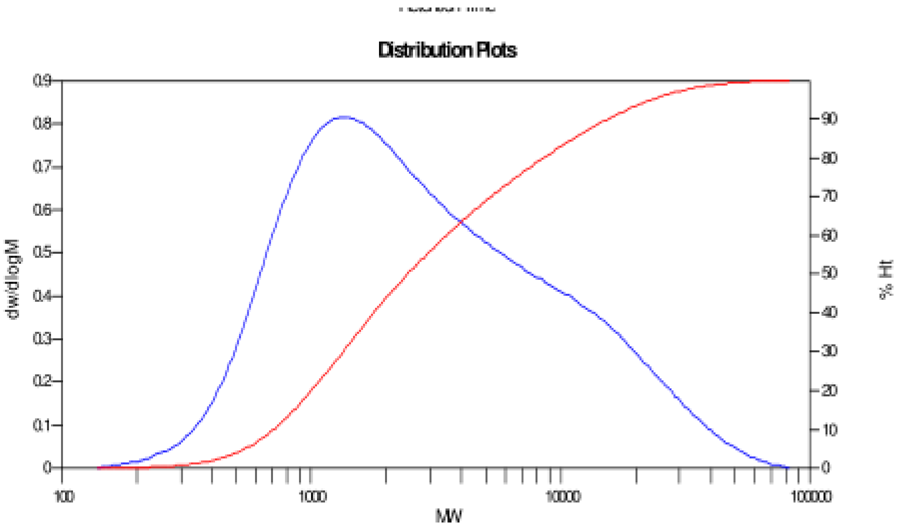

MW Averages

Peak No	Mp	Mn	Mw	Mz	Mz+1	Mv	PD
1	1386	1609	5690	17405	30115	4624	3.53636

Figure S40. GPC of the polymer from Table 1, entry 5.

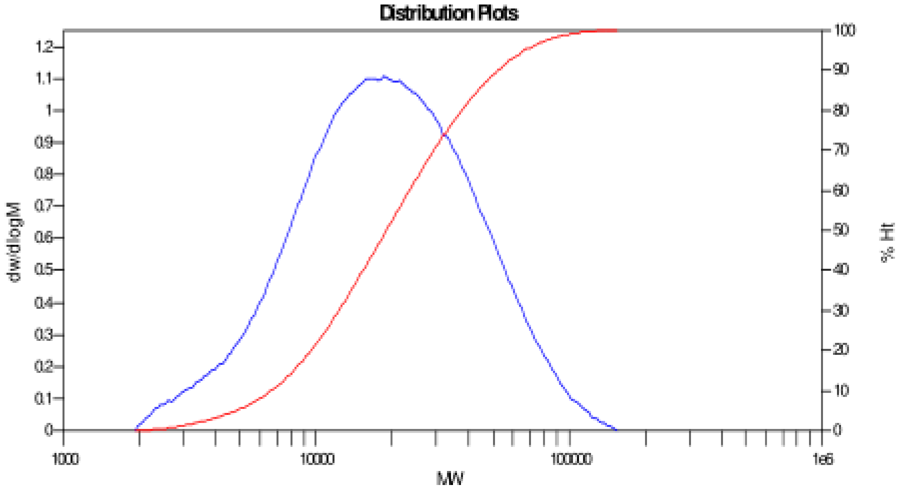

MW Averages

Peak No	Mp	Mn	Mw	Mz	Mz+1	Mv	PD
1	18518	13438	24841	41222	59198	22748	1.84856

Figure S41. GPC of the polymer from Table 1, entry 6.

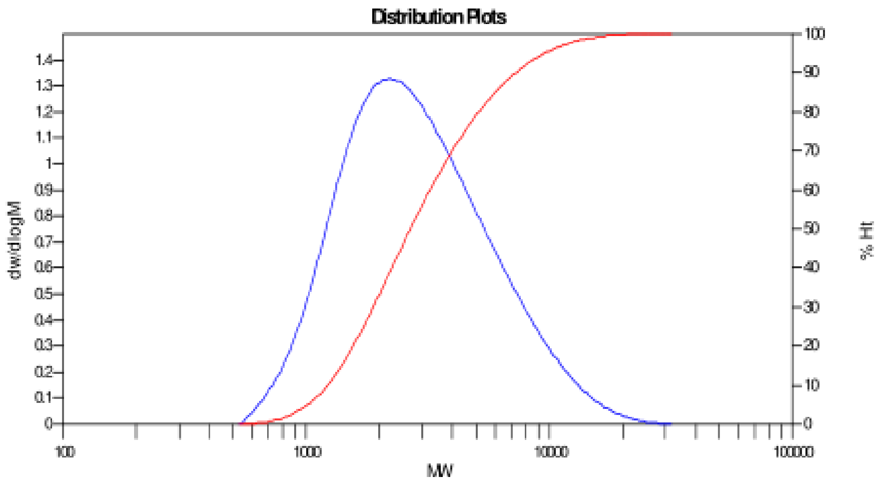

MW Averages

Peak No	Mp	Mn	Mw	Mz	Mz+1	Mv	PD
1	2205	2272	3591	5963	9229	3319	1.58055

Figure S42. GPC of the polymer from Table 1, entry 7.

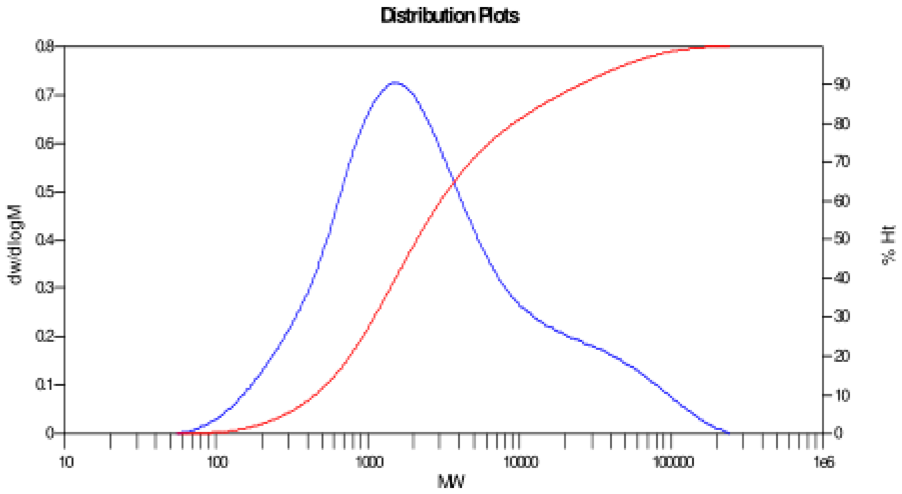

MW Averages

Peak No	Mp	Mn	Mw	Mz	Mz+1	Mv	PD
1	1488	1071	9153	55341	100086	6282	8.54622

Figure S43. GPC of the polymer from Table 1, entry 8.

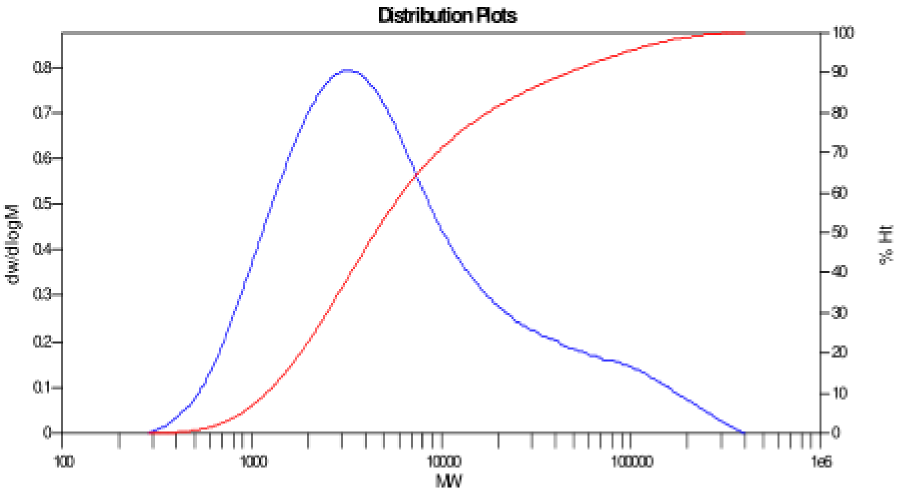

MW Averages

Peak No	Mp	Mn	Mw	Mz	Mz+1	Mv	PD
1	3157	2855	17541	98661	175974	12376	6.14396

Figure S44. GPC of the polymer from Table 1, entry 9.

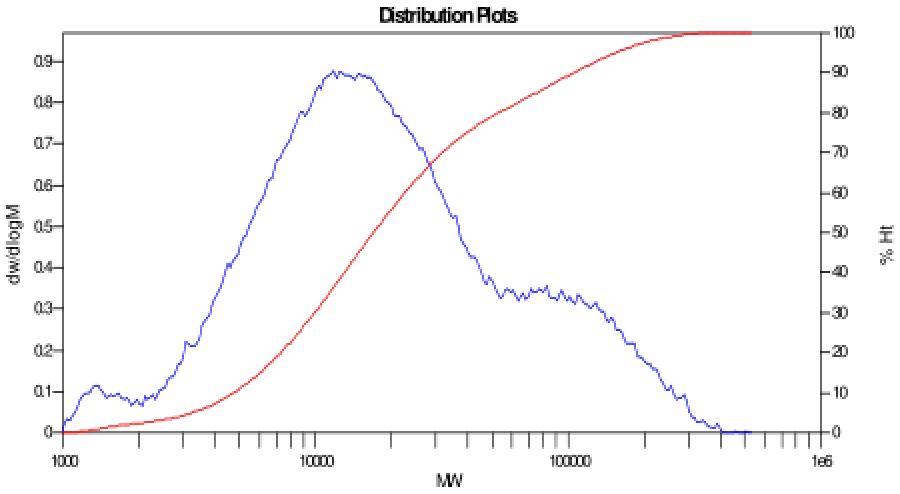

MW Averages

Peak No	Mp	Mn	Mw	Mz	Mz+1	Mv	PD
1	11817	10226	37247	108941	177575	30262	3.64238

Figure S45. GPC of the polymer from Table 1, entry 10.

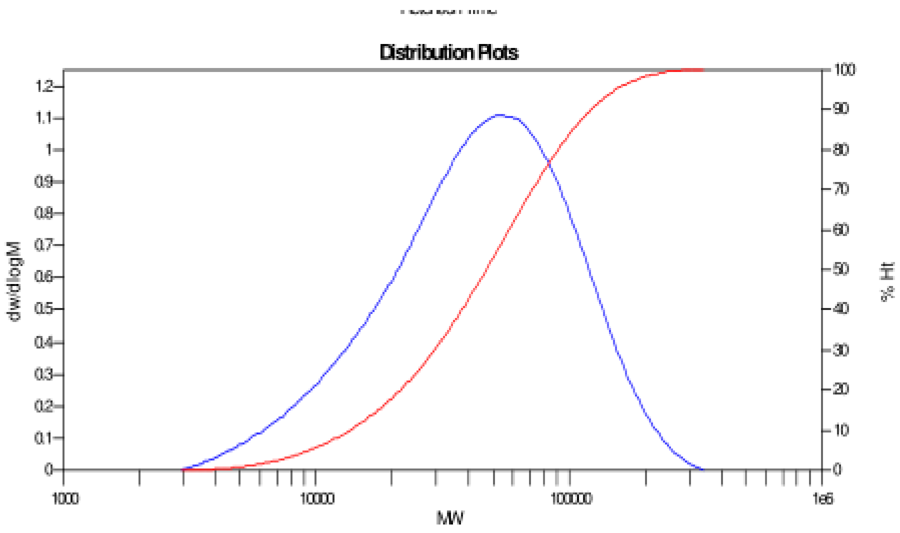

MW Averages

Peak No	Mp	Mn	Mw	Mz	Mz+1	Mv	PD
1	53663	29420	58626	94616	130405	53697	1.99273

Figure S46. GPC of the polymer from Table 1, entry 11.

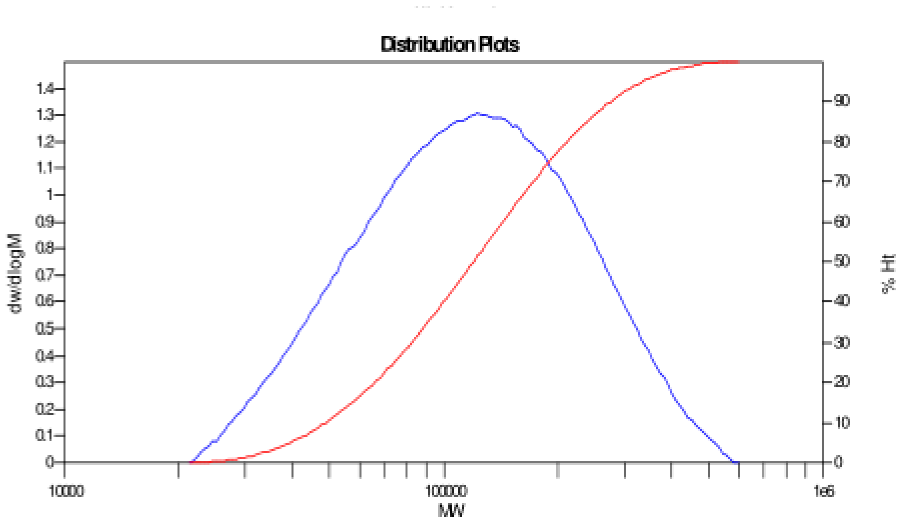

MW Averages

Peak No	Mp	Mn	Mw	Mz	Mz+1	Mv	PD
1	122760	94476	142265	202065	261866	133954	1.50583

Figure S47. GPC of the polymer from Table 1, entry 12.

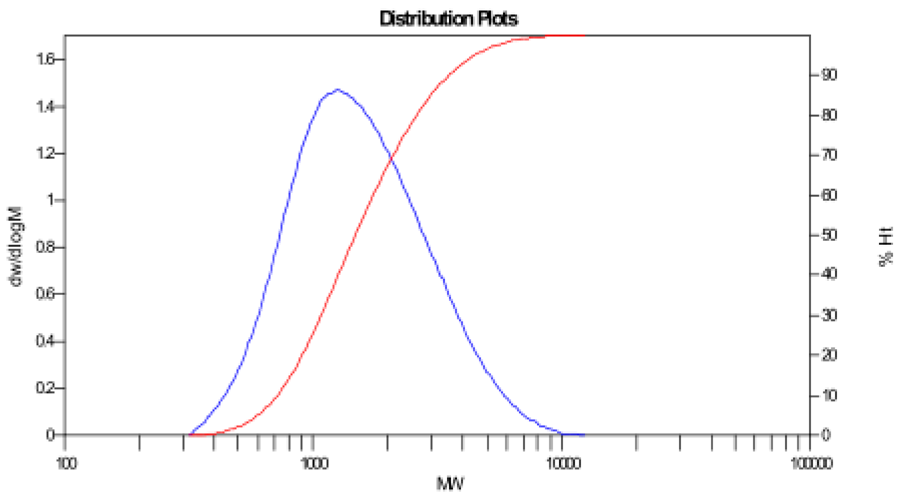

MW Averages

Peak No	Mp	Mn	Mw	Mz	Mz+1	Mv	PD
1	1243	1276	1840	2716	3845	1735	1.44201

Figure S48. GPC of the polymer from Table 1, entry 14.

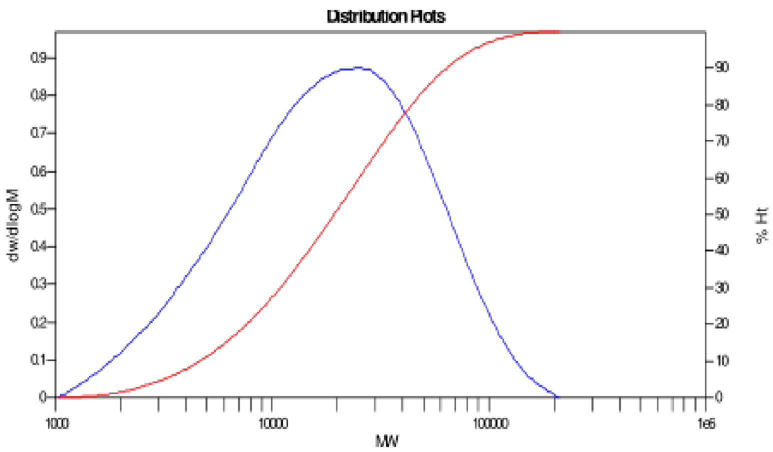

MW Averages

Peak No	Mp	Mn	Mw	Mz	Mz+1	Mv	PD
1	24019	10819	27786	53599	79998	24550	2.56826

Figure S49. GPC of the polymer from Table 1, entry 16.

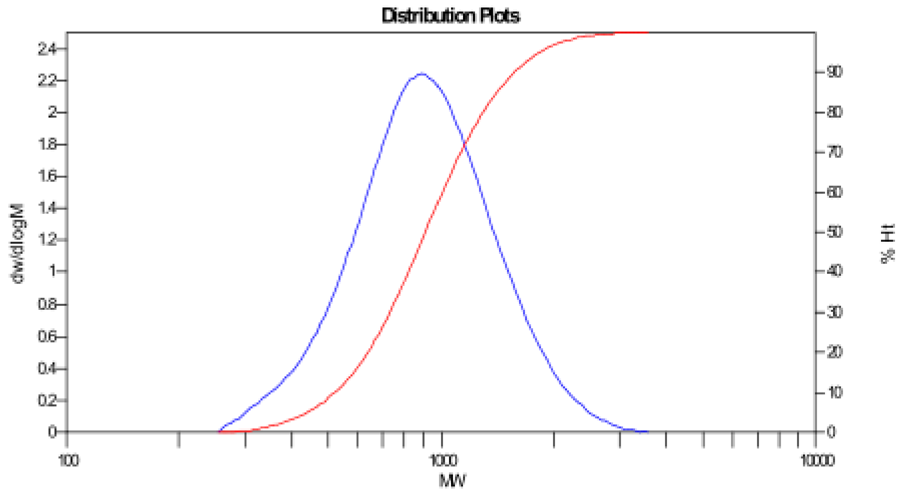

MW Averages

Peak No	Mp	Mn	Mw	Mz	Mz+1	Mv	PD
1	889	817	978	1165	1374	952	1.19706

Figure S50. GPC of the polymer from Table 2, entry 1.

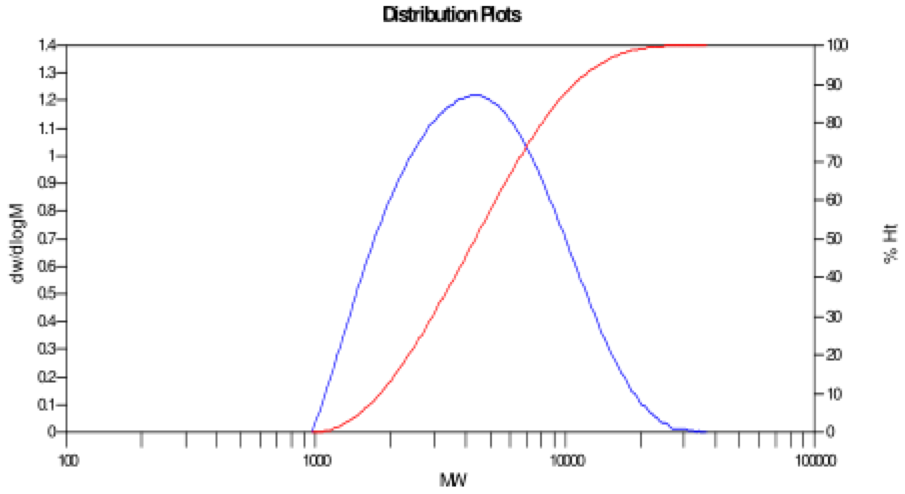

MW Averages

Peak No	Mp	Mn	Mw	Mz	Mz+1	Mv	PD
1	4275	3521	5453	8291	11545	5088	1.54871

Figure S51. GPC of the polymer from Table 2, entry 3.

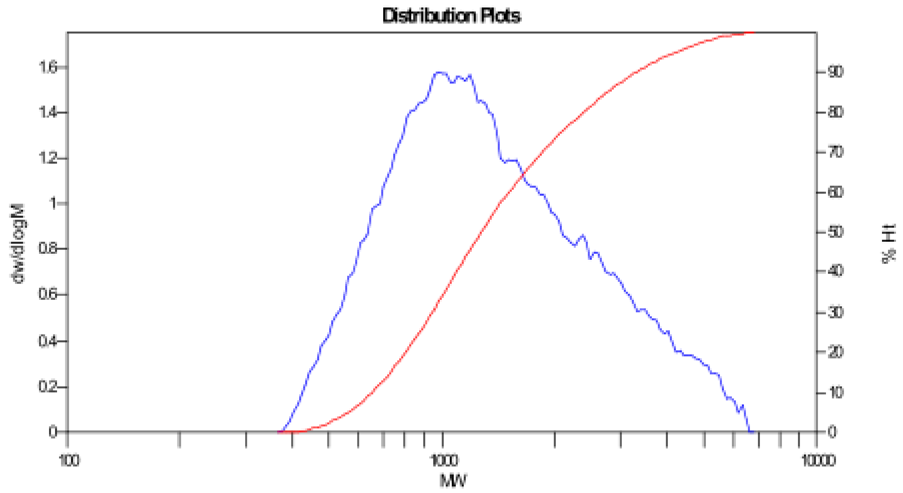

MW Averages

Peak No	Mp	Mn	Mw	Mz	Mz+1	Mv	PD
1	978	1148	1644	2415	3280	1549	1.43206

Figure S52. GPC of the polymer from Table 2, entry 4.

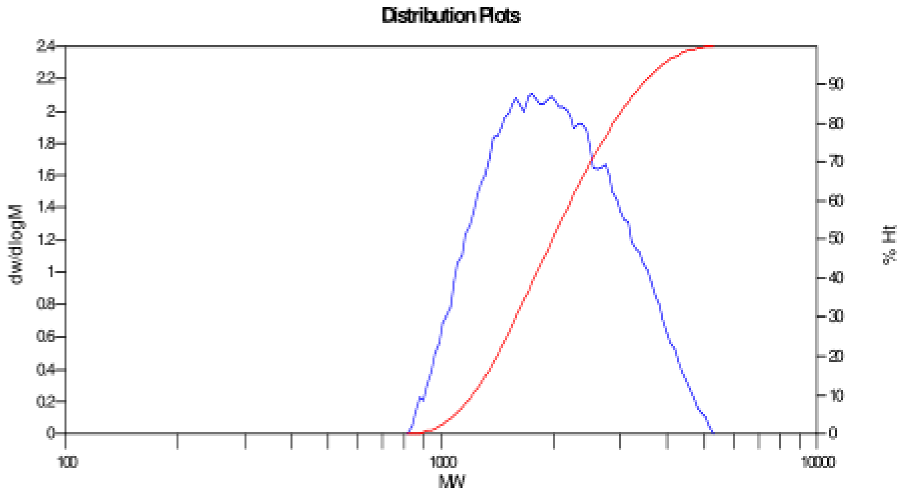

MW Averages

Peak No	Mp	Mn	Mw	Mz	Mz+1	Mv	PD
1	1741	1843	2134	2470	2816	2085	1.15789

Figure S53. GPC of the polymer from Table 2, entry 5.

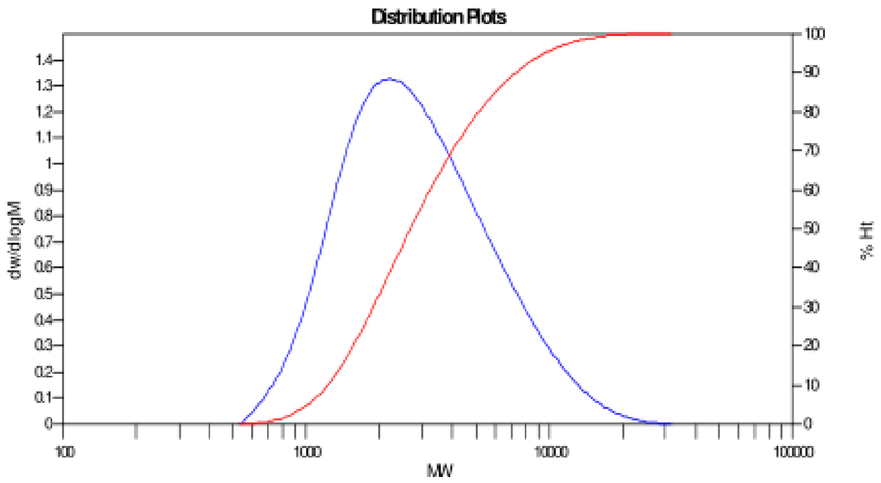

MW Averages

Peak No	Mp	Mn	Mw	Mz	Mz+1	Mv	PD
1	2205	2272	3591	5963	9229	3319	1.58055

Figure S54. GPC of the polymer from Table 2, entry 6.

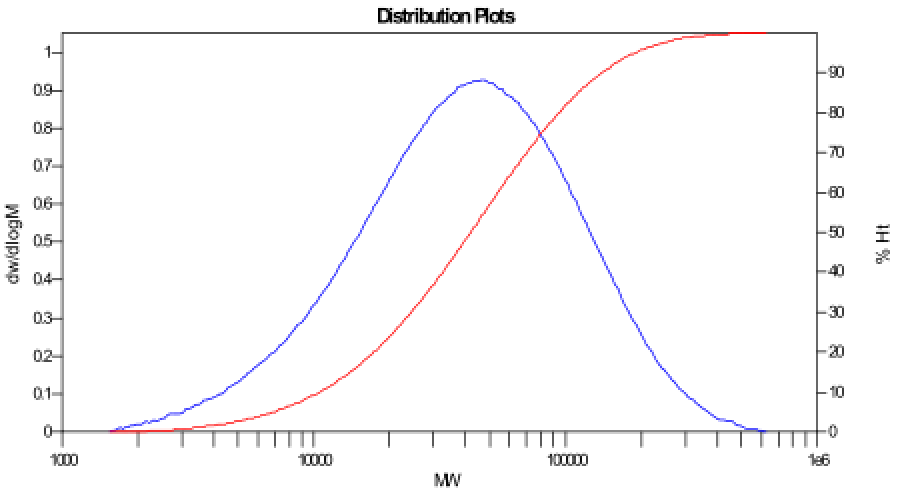

MW Averages

Peak No	Mp	Mn	Mw	Mz	Mz+1	Mv	PD
1	47399	23238	61546	126130	205084	54326	2.64851

Figure S55. GPC of the polymer from Table 2, entry 7.

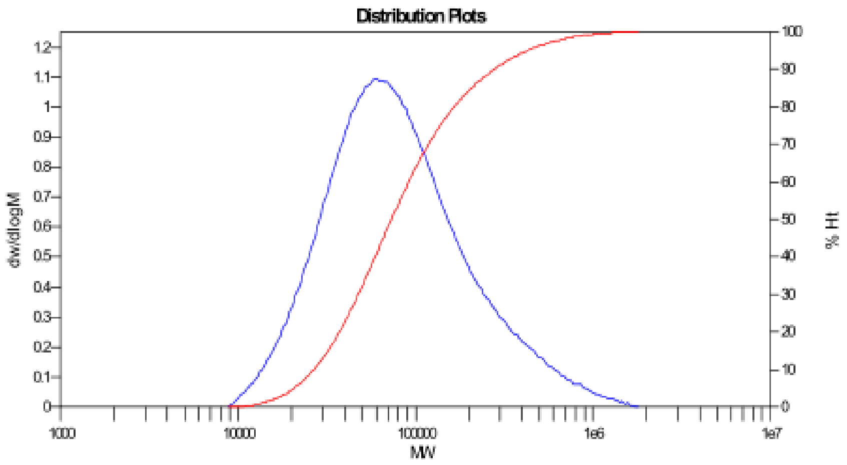

MW Averages

Peak No	Mp	Mn	Mw	Mz	Mz+1	Mv	PD
1	60228	55440	124641	327374	646251	107676	2.24821

Figure S56. GPC of the polymer from Table 2, entry 8.

5. X-Ray Crystallography of Pd2, Ni1.

Experimental for Ni1

Single crystals of $C_{46}H_{36}NiO_3P_2S$ were white. A suitable crystal was selected and mounted on a SuperNova, Dual, Cu at zero, AtlasS2 diffractometer. The crystal was kept at 291(2) K during data collection. Using Olex2 [2], the structure was solved with the ShelXT [3] structure solution program using Direct Methods and refined with the ShelXL [4] refinement package using Least Squares minimization.

Crystal structure determination of Ni1.

Crystal Data for $C_{46}H_{36}NiO_3P_2S$ (M =789.46 g/mol): triclinic, space group P-1 (no. 2), a = 9.1365(3) Å, b = 13.7655(4) Å, c = 15.5272(6) Å, α = 83.902(3)°, β = 88.918(3)°, γ = 76.670(2)°, V = 1889.44(11) Å3, Z = 2, T = 291(2) K, μ(CuKα) = 2.388 mm-1, Dcalc = 1.388 g/cm3, 12803 reflections measured (8.3° ⩽ 2Θ ⩽ 142.694°), 7121 unique (Rint = 0.0205, Rsigma = 0.0270) which were used in all calculations. The final R1 was 0.0311 (I > 2 2e used iwR2 was 0.0863 (all data).

Refinement model description

Number of restraints, 0; number of constraints, unknown.
This report has been created with Olex2, compiled on 2016.09.09 svn.r3337 for OlexSys.

Table S1. Crystal data and structure refinement for **Ni1.**

Identification code	**Ni1**
Empirical formula	$C_{46}H_{36}NiO_3P_2S$
	789.46
Formula weight	
Temperature/K	291(2)
Crystal system	triclinic

Space group	P-1
a/Å	9.1365(3)
b/Å	13.7655(4)
c/Å	15.5272(6)
α/°	83.902(3)
β/°	88.918(3)
γ/°	76.670(2)
Volume/Å^3	1889.44(11)
Z	2
ρ_{calc}g/cm^3	1.388
μ/mm^{-1}	2.388
F(000)	820.0
Crystal size/mm^3	0.250 × 0.220 × 0.210
Radiation	CuKα (λ = 1.54184)
2Θ range for data collection/°	8.3 to 142.694
Index ranges	-11 ⩽ h ⩽ 9, -16 ⩽ k ⩽ 16, -18 ⩽ l ⩽ 19
Reflections collected	12803
Independent reflections	7121 [R_{int} = 0.0205, R_{sigma} = 0.0270]
Data/restraints/parameters	7121/0/478
Goodness-of-fit on F^2	1.027
Final R indexes [I>=2σ (I)]	R_1 = 0.0311, wR_2 = 0.0846
Final R indexes [all data]	R_1 = 0.0331, wR_2 = 0.0863
Largest diff. peak/hole / e Å^{-3}	0.31/-0.43

Experimental for Pd2

Single crystals of $C_{25}H_{37}O_4PPdS_2$ **Pd2** were yellow. A suitable crystal was selected and mounted on a diffractometer. The crystal was kept at 290 ± 2 K during data collection. Using Olex2 [2], the structure was solved with the ShelXS [3] structure solution program using Direct Methods and refined with the ShelXL [4] refinement package using Least Squares minimization.

Crystal structure determination of Pd2

Crystal Data for $C_{25}H_{37}O_4PPdS_2$ (M =603.03 g/mol): monoclinic, space group C2/c (no. 15), a = 32.7912(14) Å, b = 11.1661(4) Å, c = 15.8654(5) Å, β=101.751(4)°, V = 5687.4(4) Å3, Z = 8, T = 290(2) K, μ(CuKα) = 7.386 mm-1, Dcalc = 1.409g/cm3, 20998 reflections measured (8.384° ⩽ 2Θ ⩽ 141.274°), 5373 unique (Rint = 0.1260, Rsigma = 0.0809) which were used in all calculations. The final R1 was 0.1079 (I > 2 2e used iwR2 was 0.2703 (all data).

Refinement model description

Number of restraints, 0; number of constraints, unknown.
This report has been created with Olex2, compiled on 2015.09.30 svn.r3233 for OlexSys.

Table S2. Crystal data and structure refinement for **Pd2.**

Identification code	**Pd2**
Empirical formula	C25H37O4PPdS2
Formula weight	603.03
Temperature/K	290(2)
Crystal system	monoclinic
Space group	C2/c
a/Å	32.7912(14)
b/Å	11.1661(4)
c/Å	15.8654(5)
α/°	90
β/°	101.751(4)
γ/°	90
Volume/Å3	5687.4(4)
Z	8
ϱcalcg/cm3	1.409
μ/mm-1	7.386
F(000)	2496.0
Crystal size/mm3	0.360 × 0.320 × 0.250
Radiation	CuKα (λ = 1.54184)
2Θ range for data collection/°	8.384 to 141.274
Index ranges	-39 ⩽ h ⩽ 39, -13 ⩽ k ⩽ 13, -19 ⩽ l ⩽ 13
Reflections collected	20998
Independent reflections	5373 [Rint = 0.1260, Rsigma = 0.0809]
Data/restraints/parameters	5373/0/301
Goodness-of-fit on F2	1.078
Final R indexes [I>=2σ (I)]	R1 = 0.1079, wR2 = 0.2583
Final R indexes [all data]	R1 = 0.1206, wR2 = 0.2703
Largest diff. peak/hole / e Å-3	2.16/-1.73

References

1. Ito, S.; Munakata, K.; Nakamura, A.; Nozaki, K. *J. Am. Chem. Soc.* 2009, *131*, 14606.
2. Dolomanov, O.V., Bourhis, L.J., Gildea, R.J, Howard, J.A.K. & Puschmann, H. (2009), J. Appl. Cryst. 42, 339-341.
3. G. M. Sheldrick, SHELXL 97, Programs for structure refinement, Universität Göttingen, 1997.
4. Sheldrick, G.M. (2008). Acta Cryst. A64, 112-122.
